# Targeting Adipose Tissue Function Protects Against Heart Failure with Preserved Ejection Fraction

**DOI:** 10.1002/advs.202506106

**Published:** 2025-11-23

**Authors:** Jordan Jousma, Zhenbo Han, Jooman Park, Gege Yan, Sen Zhang, Youjeong Kwon, Sarath Babu Nukala, Jindpreet Kandola, Sandra Pinho, Chong Wee Liew, Yuwei Jiang, Sang‐Ging Ong

**Affiliations:** ^1^ Department of Pharmacology & Regenerative Medicine University of Illinois College of Medicine Chicago IL 60612 USA; ^2^ Department of Physiology & Biophysics University of Illinois College of Medicine Chicago IL 60612 USA; ^3^ Division of Endocrinology Department of Medicine University of Illinois Chicago Chicago IL 60612 USA; ^4^ Department of Pharmaceutical Sciences University of Illinois Chicago Chicago IL 60612 USA; ^5^ Division of Cardiology Department of Medicine University of Illinois College of Medicine Chicago IL 60612 USA

**Keywords:** cardiometabolic, HFpEF, lipidomics, obesity, thermogenesis

## Abstract

This study seeks to develop a better understanding of how the targeting of adipose tissue (AT) can mediate outcomes in cardiac function. Obesity is highly prevalent among individuals with heart failure with preserved ejection fraction (HFpEF). While thermogenic AT helps counteract obesity‐related conditions, its impact on heart function in obesity‐related HFpEF is unclear. Using a “two‐hit” HFpEF model, the study evaluates the impact of thermogenic AT on cardiac function through pharmacological, surgical, and genetic interventions. Activation of thermogenic AT via the β3‐adrenergic receptor agonist CL‐316,243 (CL) improves cardiac function, protects against HFpEF‐induced remodeling, and enhances energy expenditure. Similarly, transplantation of AT from CL‐treated mice into wild‐type recipients confers cardioprotection. In contrast, genetic suppression of thermogenesis (*Adipoq*‐Cre; *Prdm16* ^fl/fl^) abolishes CL's benefits, while genetic enhancement of thermogenic AT (*Ucp1*‐Cre^ERT2^; *Cdkn2a* ^fl/fl^) improves cardiac structure and function. Mechanistically, AT thermogenesis is linked with significant alterations in the cardiac lipidome, as revealed by lipidomic analysis via LC/MS‐MS. These findings establish the adipose‐heart axis as a promising therapeutic target for obesity‐related HFpEF and cardiometabolic health.

## Graphical abstract

This study explores the role adipose tissue (AT) phenotypes have in determining cardiovascular outcomes in an obesity‐related heart failure with preserved ejection fraction (HFpEF) model. Pharmacological induction of thermogenesis promoted resilience to HFpEF‐induced remodeling of AT and conferred cardioprotection. Surgical and genetic models confirmed cardioprotection was mediated through AT. Mechanistically, AT targeting was linked with alterations in the cardiac lipidome.

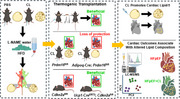

## 1 Introduction

Heart failure (HF) remains a leading cause of death and disability worldwide.^[^ ^1^ ^]^ In recent years, the prevalence of heart failure with preserved ejection fraction (HFpEF) has rapidly increased and now comprises approximately half of all HF cases. Despite having comparable mortality rates across the spectrum of HF,^[^ ^2^, ^3^, ^4^ ^]^ HFpEF individuals remain particularly vulnerable as they lack generally effective therapies, except sodium‐glucose cotransporter‐2 inhibitors (SGLT2i) and glucagon‐like peptide‐1 (GLP‐1) agonists.^[^ ^5^, ^6^, ^7^ ^]^ HFpEF is often characterized by comorbidities that lead to a systemic inflammatory state, significantly impacting cardiac function. This inflammation is believed to be a key reason why therapies directly targeting cardiovascular function have limited success. Additionally, obesity is notably common among HFpEF patients, with up to 80% reported as overweight or obese.^[^ ^8^ ^]^ Interestingly, nutrient accommodation modalities utilized during obesity appear relevant to HFpEF, as visceral but not subcutaneous adiposity is associated with an exacerbation of key functional parameters in HFpEF.^[^ ^9^, ^10^, ^11^, ^12^, ^13^ ^]^ Clinically, HFpEF is recognized as a multifactorial condition characterized by numerous and diverse sets of comorbidities, including conditions such as renal insufficiency and pulmonary hypertension.^[^ ^14^, ^15^ ^]^ The identification of distinct phenogroups within the HFpEF population, such as those characterized by obesity, emphasizes the need for tailored animal models to accurately represent the diverse pathological sources of dysfunction.^[^ ^16^, ^17^, ^18^ ^]^ To address this, the “two‐hit” HFpEF mouse model developed by others^[^ ^19^ ^]^ incorporates metabolic and hypertensive stress through a high‐fat diet (HFD) and *N* ^ω^‐nitro‐L‐arginine methyl ester (L‐NAME) in drinking water, providing a more relevant framework for studying the multifactorial nature of HFpEF, particularly in the context of obesity.

Adipose tissue (AT) has become increasingly recognized as a highly dynamic organ with diverse metabolic and endocrine functions, including the regulation of nutrient storage and the circulation of biologically active lipid species. When challenged by obesogenic diets, AT can adapt to handle excess nutrients, achieving healthy accommodations up to a certain limit.^[^ ^20^, ^21^, ^22^, ^23^, ^24^ ^]^ One of the most notable adaptations is thermogenesis, where adipocytes shift from storing energy to engaging in futile nutrient cycling to generate heat.^[^ ^25^ ^]^ This process is primarily mediated through mitochondrial uncoupling protein 1 (UCP1), which allows proton diffusion across the inner mitochondrial membrane, decoupling the electrochemical gradient from ATP synthesis.^[^ ^26^ ^]^ Previously, thermogenesis was thought to occur only in infancy, but it is now recognized as occurring in adult humans, particularly following treatment with a β3‐adrenergic receptor (β3‐AR) agonist or cold environment.^[^ ^27^, ^28^, ^29^ ^]^ This discovery reinvigorated interest in the role of thermogenic AT in human health. In the context of HFpEF, thermogenic AT has been associated with favorable cardiometabolic traits in humans.^[^ ^30^ ^]^ Conversely, obesity and aging, both prevalent in HFpEF, are associated with reduced thermogenic capacity in AT.^[^ ^31^, ^32^, ^33^, ^34^, ^35^ ^]^ Experimentally, thermogenic AT is beneficial against obesity‐related dysfunction.^[^ ^36^, ^37^, ^38^ ^]^ Although thermogenic AT has been previously noted in an HFpEF model,^[^ ^39^ ^]^ its specific role in an obesity‐related HFpEF model remains to be fully understood.

Herein, we utilized pharmacological, surgical, and genetic models to investigate the role of AT in regulating cardiac function in HFpEF. Our findings demonstrate that the β3‐AR agonist CL‐316243 (CL) exerts a cardioprotective role in obesity‐related HFpEF, likely due to the result of targeting AT rather than a direct effect on the heart. Transplantation of CL‐treated AT conferred similar cardioprotection, directly implicating AT as the key target of CL. Using a genetic model (*Adipoq*‐Cre; *Prdm16* ^fl/fl^) where induction of thermogenesis is impaired, we found that the CL‐mediated cardioprotective was largely abolished. Conversely, a thermogenic AT‐specific model (Ucp1‐Cre^ERT2^; Cdkn2a^fl/fl^) with enhanced AT thermogenesis improved cardiac structure and function in HFpEF.^[^ ^40^ ^]^ Finally, we demonstrated that thermogenesis modulates cardiac lipid composition and used LC/MS‐MS to characterize the cardiac lipidome, revealing significant differences between HFpEF and the protected state. In summary, our findings reveal that AT influences cardiac outcomes in HFpEF, likely by shaping the cardiac lipidome. These results suggest that enhancing AT thermogenesis could be a promising strategy for developing new HFpEF therapies.

## 2 Results

### 2.1 Pretreatment with CL‐316243 is Cardioprotective Against HFpEF‐Induced Dysfunction

**Figure 1:**
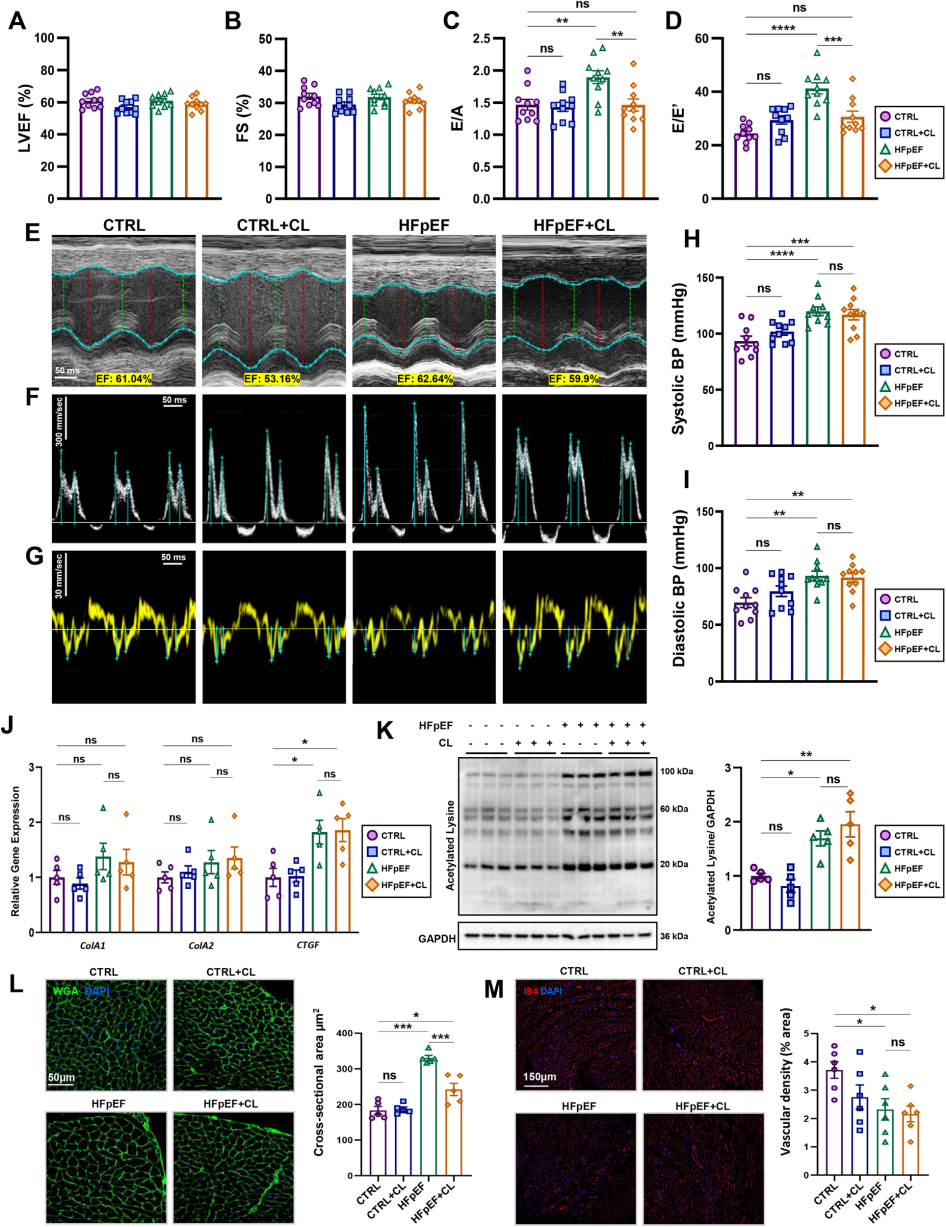
Pretreatment with CL is cardioprotective against diastolic dysfunction and cardiomyocyte hypertrophy. A) Measurements of left ventricular ejection fraction (LVEF) and B) fractional shortening (FS) were determined by transthoracic echocardiography. Pulsed‐wave Doppler and tissue Doppler measurements recorded from the apical four‐chamber views were used to calculate the diastolic parameters C) E/A and D) E/E’; *n* = 10 mice per group. E–G) Representative images from m‐mode, pulsed‐wave, and tissue Doppler mode recordings for each of the indicated groups; scale bars indicate time (x‐axis) of 50 ms and velocity (y‐axis) of 300 mm sec^−1^. H,I) Blood pressure measurements were obtained using the CODA tail‐cuff monitoring system, measuring systolic and diastolic pressures recorded in acclimatized mice; *n* = 10 mice per group. J) Gene expression analysis was measured by RT‐qPCR, evaluating the expression of fibrosis‐related genes (*Col1a1*, *Col1a2*, and *Ctgf*) in cardiac extracts; *n* = 5 per group. K) Western blot result obtained using an acetylated lysine antibody in cardiac extracts; *n* = 5 per group. L) Representative images of immunofluorescent staining of wheat germ agglutinin stained hearts and quantification of cardiomyocyte cross‐sectional area; *n* = 5 per group. M) Isolectin‐IB4‐stained hearts with quantification of the percentage of the positively stained area per field of view; *n* = 6 per group. Scale bars are equal to 50 µm (WGA) or 150 µm (IB4). Data are presented as the mean ± SEM; significance values were determined by one‐way analysis of variance (ANOVA) followed by Tukey's multiple comparisons. *P ≤ 0.05, **P ≤ 0.01, ***P ≤ 0.001, ****P ≤ 0.0001.

To better understand the role of thermogenic adipose tissue (AT) in heart failure with preserved ejection fraction (HFpEF), we first utilized a pharmacological approach to activate AT before the onset of HFpEF, and then employed an obesity‐related two‐hit HFpEF model developed by others.^[^ ^19^ ^]^ AT activation was achieved with the β3‐adrenergic receptor (β3‐AR) agonist CL‐316243 (CL), administered at a dose of 1 mg/kg/day via intraperitoneal injection for seven days.^[^ ^41^ ^]^ Following CL treatment, mice were subjected to HFpEF‐inducing conditions for seven weeks (Figure S1A, Supporting Information). CL treatment transiently affected body weight with the induction of thermogenesis; however, a recovery in the following weeks occurred, and by the end of HFpEF treatment, no significant differences were observed in body weight between HFpEF and HFpEF+CL groups (Figure S1B,C, Supporting Information). Cardiac function was then measured using transthoracic echocardiography, which showed that left ventricular ejection fraction (LVEF) and fractional shortening (FS) remained comparable across all groups (**Figure** **1**A,B). Parameters of diastolic dysfunction commonly recognized in this model,^[^ ^42^ ^]^ including an elevated E/A and E/E’ were also measured. Notably, HFpEF+CL mice were protected from diastolic dysfunction (Figure 1C,D), as illustrated by representative tracings (Figure 1E,G). HFpEF mice also showed a significant increase in calculated LV mass, indicating hypertrophic remodeling. In contrast, HFpEF+CL mice had a significantly reduced LV mass compared to the HFpEF group, suggesting reduced hypertrophy, while additional echocardiographic parameters remained unchanged across different treatment groups (Table S1, Supporting Information). Blood pressure measured via a tail‐cuff monitoring system indicated that CL treatment did not alleviate elevated pressures (Figure 1H,I; Figure S1D, Supporting Information). The extent of CL‐mediated cardioprotection was further characterized in HFpEF+CL mice by reductions in cardiac hypertrophy and pulmonary congestion, as measured by heart weight‐to‐tibia length and total lung water, both of which were significantly attenuated in HFpEF+CL mice (Figure S1E,F, Supporting Information).

Additional pathological features reported in this model, including fibrosis, protein hyperacetylation, cardiomyocyte hypertrophy, and vascular rarefaction, were also assessed.^[^ ^19^ ^]^ Picrosirius red staining revealed significant increases in perivascular fibrosis in both HFpEF and HFpEF+CL mice, while interstitial fibrosis appeared to be unaffected across all groups (Figure S1G,H, Supporting Information). Accordingly, the pro‐fibrotic gene *Ctgf* expression was significantly increased in both HFpEF and HFpEF+CL (Figure 1J). Cardiac protein hyperacetylation, a previously described feature of cardiometabolic HFpEF,^[^ ^43^ ^]^ also appeared to be unmitigated by CL treatment (Figure 1K). In contrast, cardiomyocyte hypertrophy, determined by wheat germ agglutinin (WGA) staining, showed improvements in HFpEF+CL mice compared to HFpEF mice (Figure 1L). Finally, vascular rarefaction, as assessed by Isolectin gs‐ib4 (IB4) staining, revealed that both HFpEF and HFpEF+CL mice experienced a similar reduction in vascular density, with significant decreases relative to CTRL mice (Figure 1M).

Overall, these findings characterize the cardioprotective effects of CL treatment, including improvements in diastolic function, pulmonary congestion, and hypertrophic remodeling. At the same time, other pathological manifestations, such as those affecting the vasculature, appeared to be unmitigated. This may reflect how stressors such as L‐NAME can disproportionally affect cell types that are particularly sensitive to nitric oxide bioavailability, such as endothelial cells, for instance.^[^ ^44^ ^]^ Importantly, CL treatment did not produce any lasting effects on cardiac function under basal conditions, as no significant differences were observed between CTRL+CL and CTRL in any parameter. This suggests that while CL exerts cardioprotective effects specifically in the context of HFpEF, it has minimal residual impact on cardiac function under normal conditions.

### 2.2 CL‐Mediated Cardioprotection is not Directly Linked to the Activation of the Cardiac β3‐AR Pathway

**Figure 2:**
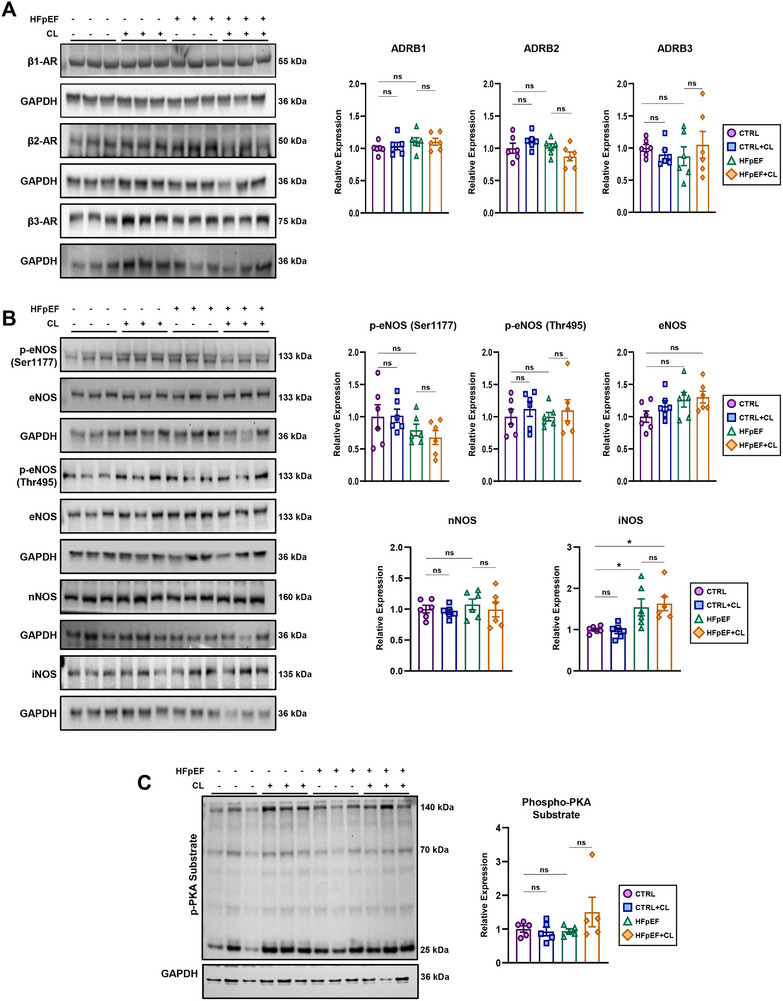
Molecular signature of β3‐adrenergic signaling in the heart. A) Western blotting of cardiac extracts to determine the expression of β‐adrenergic receptors (β1‐3); *n* = 6 per group. B) Western blotting of nitric oxide synthases and phosphorylation status at phospho‐active/ inhibitory residues; *n* = 6 per group. C) PKA activity as measured by the total amount of phosphorylated PKA substrates in cardiac protein lysates determined by western blotting; *n* = 5 per group. Data are presented as the mean ± SEM; significance values were determined by one‐way analysis of variance (ANOVA) followed by Tukey's multiple comparisons. *P ≤ 0.05, **P ≤ 0.01, ***P ≤ 0.001, ****P ≤ 0.0001.

Under basal conditions, murine cardiomyocytes appear to have minimal expression of the β3‐AR and lack the ability to respond to CL treatment.^[^ ^45^, ^46^ ^]^ However, in failing hearts, expression may increase,^[^ ^47^ ^]^ and in mice, this receptor is known to mediate potentially relevant downstream effects.^[^ ^48^ ^]^ Thus, we sought to address whether CL‐mediated cardioprotection might result from direct targeting of the heart. We first evaluated the expression of β‐ARs since they may be downregulated following activation.^[^ ^49^ ^]^ Although CL has >10^5^‐fold selectivity for the β3‐AR over the β1 or β2,^[^ ^50^ ^]^ we included all three β‐Ars to rule out potential off‐target activity. Gene and protein expression levels remained unaltered across all groups, indicating the absence of ligand‐induced AR downregulation (**Figure** **2**A; Figure S2A, Supporting Information). In cardiac tissues, β3‐AR signaling is coupled to the activation of nitric oxide synthases (NOS) and cyclic guanosine monophosphate (cGMP) production, which can subsequently influence cyclic adenosine monophosphate (cAMP) and protein kinase A (PKA) activity.^[^ ^48^ ^]^ Western blot analysis of NOS, including both active (Ser1177) or inhibitory (Thr495) residues of eNOS, as well as total eNOS and nNOS, revealed no significant changes. Expression of iNOS, however, was significantly increased, consistent with prior reports of this HFpEF model^[^ ^19^ ^]^ (Figure 2B). PKA activity, assessed using a pan p‐PKA substrate antibody, revealed no significant changes across groups (Figure 2C). Similarly, competitive ELISA measurements of cGMP and cAMP levels in cardiac extracts showed no significant alterations (Figure S2B, Supporting Information).

While these results indicated that neither HFpEF nor CL had a lasting effect on this pathway, we also recognized the importance of assessing an earlier time point where the effects of CL may be more pronounced. Thus, a similar set of experiments was performed immediately following a course of CL treatment but before the start of HFpEF treatment. Expression levels of β1‐3‐AR remained comparable across groups, and both active and inhibitory residues of eNOS and nNOS showed no significant changes (Figure S2C, Supporting Information). There was, however, a slight, albeit significant, increase in the expression of iNOS (Figure S2C, Supporting Information). Downstream signaling involving PKA activity appeared unchanged after CL treatment (Figure S2D, Supporting Information). Overall, these findings suggest that the heart is relatively unresponsive to direct stimulation by CL under basal conditions, aside from iNOS upregulation. Although typically associated with being a feature of inflammation, it is important to note that the downstream effects mediated by iNOS are also known to serve a variety of beneficial roles.^[^ ^51^ ^]^ Thus, the cardioprotective effects of CL do not appear to result from direct targeting of cardiomyocytes. Accordingly, under basal conditions, we isolated cardiac endothelial cells and cardiomyocytes to examine the distribution of the β3‐AR and observed that expression was primarily restricted to endothelial cells (Figure S2E, Supporting Information). However, aspects of vascular health (rarefaction and hypertensive stress) did not appear to improve in HFpEF+CL mice (Figure 1H,I,M), suggesting cardioprotection originates from outside the heart.

### 2.3 Pharmacologically Induced Thermogenic Adipose Tissue is Resilient to HFpEF‐Induced Adipose Remodeling

Since CL did not appear to act directly on the heart, we examined whether its cardioprotective effects originated from AT, a known primary target of β3‐AR agonists. While cardiac tissue showed minimal acute response to CL treatment, AT, by contrast, underwent significant changes, as evidenced by immunohistochemical (IHC) staining for uncoupling protein 1 (UCP1) of interscapular brown (iBAT), inguinal white (iWAT), and gonadal white (gWAT) (Figure S3A, Supporting Information). These results suggest that AT is more responsive than cardiac tissue to CL and may serve as the primary mediator of its cardioprotective effects.

**Figure 3:**
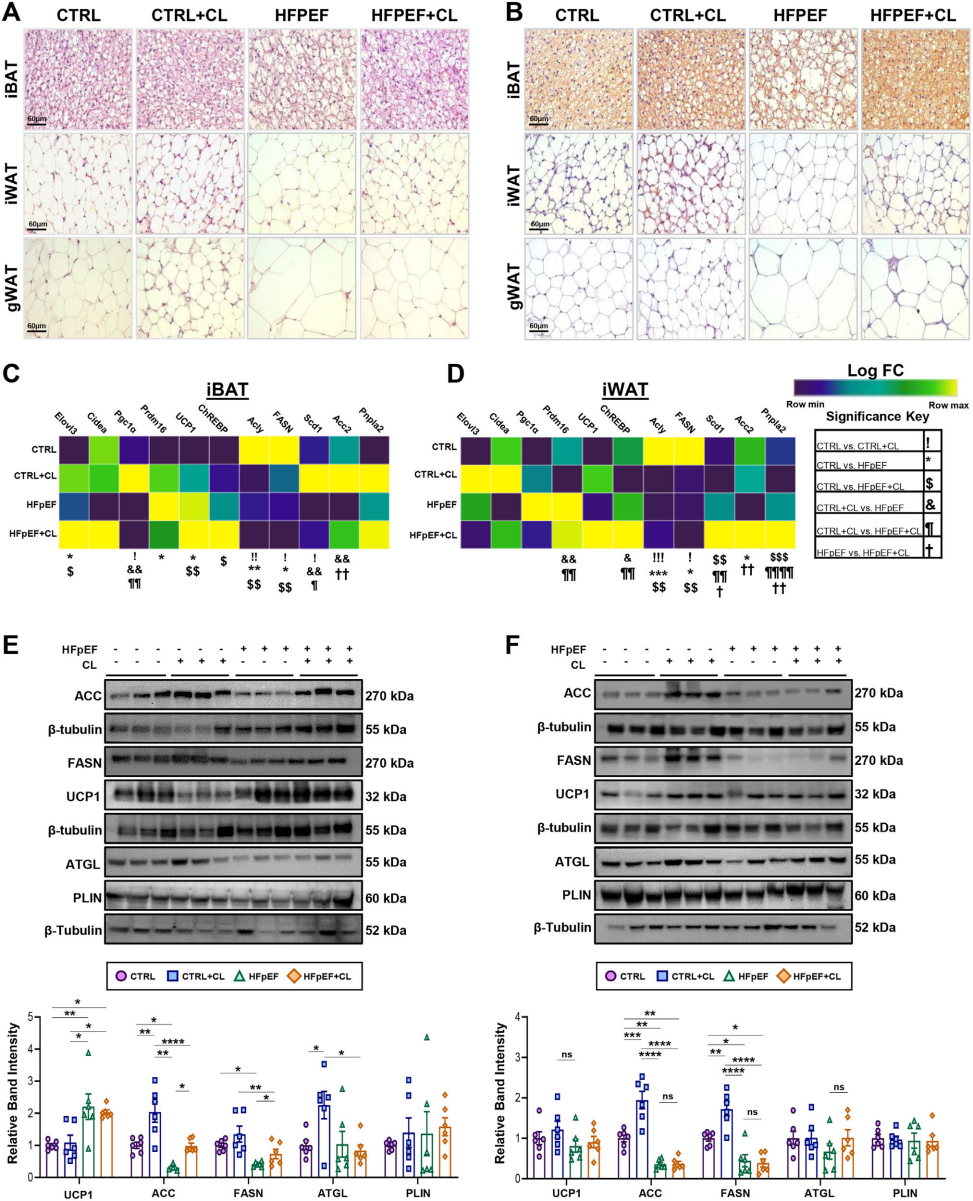
Histological and molecular characterizations of adipose tissues. A) Hematoxylin and eosin (H&E) staining of interscapular brown adipose tissue (iBAT), inguinal white adipose tissue (iWAT), and gonadal white adipose tissue (gWAT) of either CTRL, CTRL+CL, HFpEF, or HFpEF+CL groups. B) Immunohistochemical staining of UCP1 in iBAT, iWAT, and gWAT; image magnifications are recorded using 40x objective; scale bars equal 60 µm. The LogFC in gene expression for a panel of thermogenic markers determined by RT‐qPCR is colored according to the gradient displayed in the legend, while symbols for significance values correspond to those shown in the figure legend showing changes in the C) iBAT and D) iWAT; *n* = 6 per group. Western blot results for thermogenic markers and enzymes involved in de novo lipogenesis and lipolysis in E) iBAT and F) iWAT; *n* = 6 per group. Data are presented as the mean ± SEM; significance values were determined by one‐way analysis of variance (ANOVA) followed by Tukey's multiple comparisons. *P ≤ 0.05, **P ≤ 0.01, ***P ≤ 0.001, ****P ≤ 0.0001.

To assess whether CL treatment had lasting effects on AT, we examined histological changes in iBAT, iWAT, and gWAT using hematoxylin and eosin (H&E) staining (**Figure** **3**A; Figure S3B, Supporting Information) along with IHC staining of UCP1 (Figure 3B; Figure S3C, Supporting Information). This revealed that HFpEF+CL mice were able to resist HFpEF‐induced iBAT whitening and exhibited reduced hypertrophic expansion of white adipocytes. Given that weight gain appeared to be unaffected by CL treatment (Figure S1B, Supporting Information), this suggests a “healthier” mode of nutrient accommodation, involving adipogenesis that occurred in the AT of HFpEF+CL mice.^[^ ^21^ ^]^

We next examined the expression of key thermogenic markers using RT‐qPCR, focusing on iBAT and iWAT due to their high thermogenic capacity. Among transcriptional regulators in iBAT (*ChREBP*, *Cidea*, *Prdm16*, *Pgc1α*), *Prdm16* was significantly upregulated in HFpEF mice, while *ChREBP* was elevated in HFpEF+CL mice compared to CTRL (Figure 3C). Among de novo lipogenesis (DNL) genes (*Acly*, *Fasn*, *Scd1*, *Acc2*), HFpEF treatment exerted a broadly suppressive effect, with both HFpEF and HFpEF+CL experiencing significant reductions in *Acly* and *Fasn* in the iBAT and iWAT compared to CTRL mice. However, in the iWAT of HFpEF+CL, the expression of *Scd1*, *Acc2*, and *Pnlpa2* significantly increased compared to HFpEF mice (Figure 3D). Yet, downstream signaling events of β3‐AR signaling remained unchanged across all groups, as indicated by PKA activity determined by western blotting (Figure S3D,E, Supporting Information). Thus, only a subset of genes involved in thermogenesis appeared to have effects that persisted throughout HFpEF treatment.

Interestingly, a form of metabolic conditioning known as “thermogenic memory,” where lasting metabolic adaptations, such as increased energy expenditure, have recently been shown to depend on sustained expression of DNL enzymes.^[^ ^52^ ^]^ Thus, we sought to address how proteins involved in DNL and lipolysis, as both processes are known to have an important role in supporting thermogenic functions. We first observed increased UCP1 expression in the iBAT (Figure 3E) but not the iWAT (Figure 3F) of HFpEF and HFpEF+CL mice compared to CTRL mice, which is known to occur with HFD feeding.^[^ ^53^ ^]^ As expected, ACC and FASN expression were significantly downregulated in HFpEF compared to CTRL in both depots (Figure 3E,F). In CL‐treated mice, though, expression of ACC and FASN was increased. In the iBAT, expression of ACC and FASN was notably increased in HFpEF+CL mice compared to HFpEF mice (Figure 3E). Similarly, CTRL+CL mice also had significantly increased expression of ACC and FASN compared to CTRL mice in both the iBAT and iWAT (Figure 3E,F). However, lipolytic regulators (ATGL, PLIN) showed no significant differences between HFpEF and HFpEF+CL mice. As AT lipolysis is known to produce changes in circulating metabolites,^[^ ^54^ ^]^ we also conducted serum analysis of non‐esterified fatty acids (NEFA) and beta‐hydroxybutyrate (BHB) to evaluate if systemic indicators of this activity could be resolved (Figure S3F,G, Supporting Information). There were no significant increases in either NEFA or BHB to indicate an increase in lipolytic activity. However, HFpEF mice did appear to suffer from a significant reduction in serum BHB compared to all other groups.

Immunometabolic regulation of the AT by resident and migratory immune cells is known to affect systemic metabolic functions.^[^ ^55^ ^]^ To address this potential link, we profiled the peripheral blood (PB) and AT using Fluorescence‐activated cell sorting (FACS). In the PB, CL‐treated mice were shown to have an elevated CD4:CD8 ratio that appeared to be driven by increases in CD4+ cells (Figure S4A, Supporting Information). In HFpEF, there was a significant increase in proinflammatory Gr1+ Mac‐1+ cells (G+M+) cells, which are known to expand during chronic inflammation and obesity.^[^ ^56^, ^57^ ^]^ In HFpEF+CL mice, though, G+M+ cell count was significantly reduced compared to HFpEF mice, indicating an improvement in this aspect of systemic inflammation (Figure S4A, Supporting Information). In the AT, CD11c and CD206 were used to differentiate between M1 and M2 macrophages. While there were no significant changes in the iBAT, analysis of iWAT revealed that there was a significant reduction in the amount of proinflammatory CD11c+ CD206+ double‐positive macrophages in HFpEF+CL mice compared to HFpEF mice (Figure S4B, Supporting Information). In the gWAT, CD11c^−^ CD206^−^ double negative macrophages, which have anti‐inflammatory roles in obese AT,^[^ ^58^ ^]^ were significantly increased in both CTRL+CL and HFpEF+CL when compared to HFpEF mice (Figure S4B, Supporting Information).

Overall, these results show how thermogenic conditioning of AT leads to divergent forms of AT remodeling when challenged by the stressors of HFpEF treatment. These enduring effects appeared to promote a healthier form of AT expansion, including the ability to resist iBAT whitening and suppression of DNL enzymes induced by HFpEF, while also tempering immunologically derived indicators of inflammation.

### 2.4 CL Treatment Enhances Metabolic Functions Impaired by HFpEF

**Figure 4:**
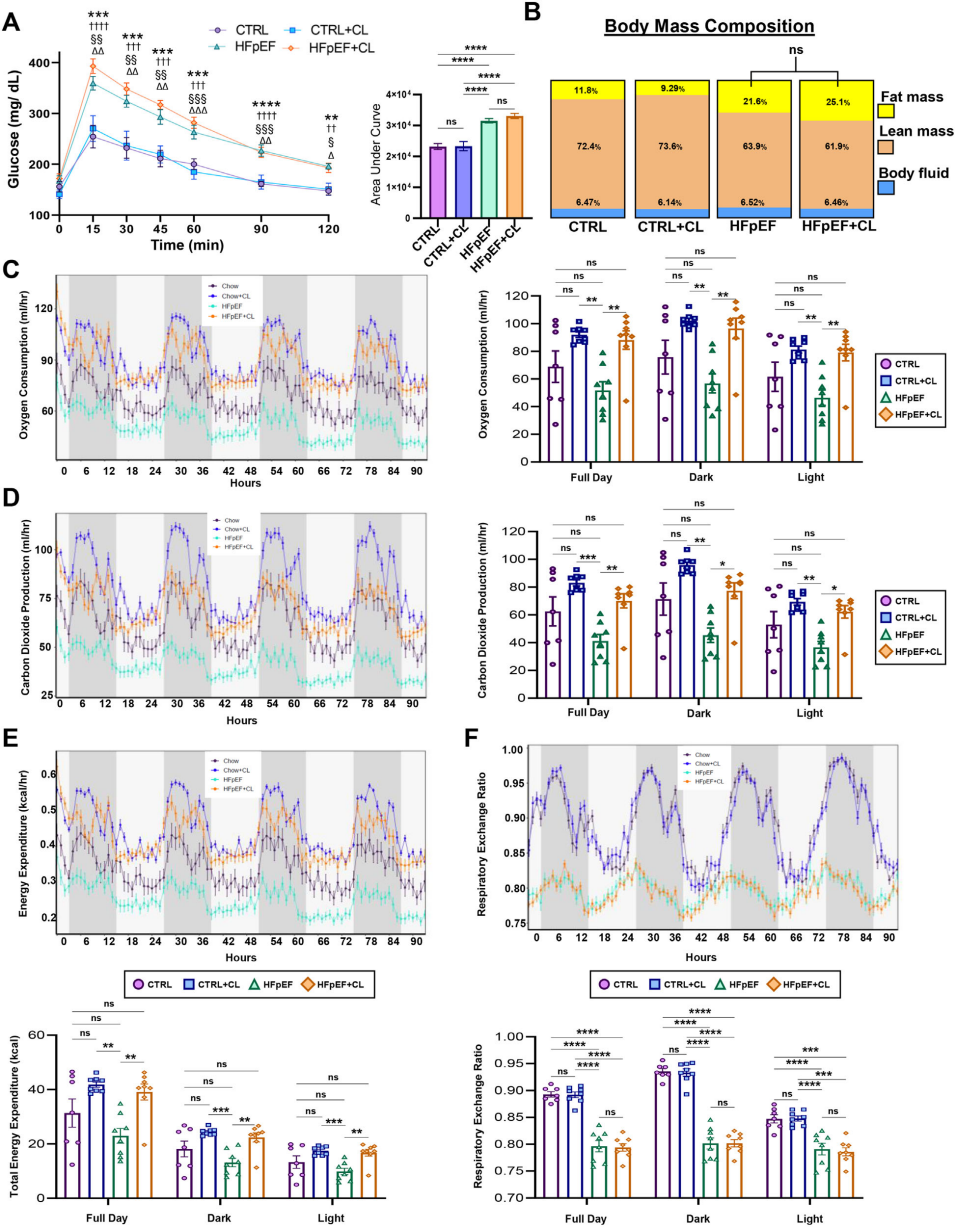
CL treatment enhances metabolic functions, which deteriorate with the induction of HFpEF. Baseline measurements of fasting blood glucose levels and the values in blood glucose following administration of intraperitoneal bolus injection of glucose solution were recorded at the indicated time points. A) The corresponding area under the curve plots shows how glucose tolerance was affected by each treatment. B) Body mass compositions were determined by nuclear magnetic resonance (NMR); *n* = 12 mice per group. Indirect calorimetry measurements obtained from metabolic chambers determined C) oxygen consumption, D) carbon dioxide production, E) energy expenditure, and F) respiratory exchange ratio; *n* = 8 mice per group. Data are reported in line charts over the indicated time periods displayed in hours, with light and dark periods shaded accordingly, while box plots display the aggregate values obtained over a defined period of activity (full day, dark, light). Data are reported as the mean ± SEM; significance values were determined by a one‐way analysis of variance (ANOVA) followed by Tukey's multiple comparisons. *P ≤ 0.05, **P ≤ 0.01, ***P ≤ 0.001, ****P ≤ 0.0001. The significance key for GTT is as follows: *=CTRL versus HFpEF; †=CTRL versus HFpEF+CL; §= CTRL+CL versus HFpEF; ∆=CTRL+CL versus HFpEF+CL, which describes values determined by a one‐way analysis of variance (ANOVA) followed by Tukey's multiple comparisons taken at each indicated time point.

We hypothesized that the AT phenotypes outlined above may be linked with metabolic adaptations. Since HFpEF is a systemic condition, we assessed whole‐body metabolic function, as is commonly done to evaluate thermogenic activity. During the acute phase following CL treatment, AT is known to readily uptake glucose.^[^ ^59^ ^]^ However, the glucose tolerance test (GTT) revealed no CL‐specific effects. Instead, glucose tolerance was significantly impaired for both HFpEF and HFpEF+CL, while CTRL and CTRL+CL mice retained normal glucose sensitivity (**Figure** **4**A). Similarly, body mass composition, determined by nuclear magnetic resonance (NMR), showed no CL‐specific effects regarding the distribution of fat or lean mass (Figure 4B).

We next examined whole‐body metabolism using indirect calorimetry. Here, we observed an HFpEF‐specific effect as well as a CL‐specific effect. CTRL+CL and HFpEF+CL mice significantly increased O_2_ and CO_2_ consumption compared to HFpEF but not CTRL mice (Figure 4C,D). This pattern was mirrored in energy expenditure (EE), where both CTRL+CL and HFpEF+CL groups showed significantly increased EE compared to HFpEF mice (Figure 4E). Importantly, these EE differences could not be attributed to changes in food or water intake or locomotor activity, as none of these parameters followed the same trend (Figure S5A–C, Supporting Information). The respiratory exchange ratio (RER) indicated that HFpEF severely impairs metabolic flexibility. Both HFpEF and HFpEF+CL mice exhibited a pronounced reliance on fatty acid oxidation (FAO), whereas CTRL and CTRL+CL mice maintained normal substrate oscillations, including glucose oxidation, aligned with the light‐dark cycle (Figure 4F). This suggests that the elevated EE in HFpEF+CL mice is primarily driven by lipid metabolism.

Next, we performed a regression analysis to evaluate the relationship between the NMR‐measured body mass composition and EE. The total mass and lean mass, regarded as more metabolically active tissues, tended to have positive correlations with EE for all groups except in HFpEF mice. For HFpEF mice, lean mass appeared to have the weakest correlation with energy expenditure (Figure S5D,E, Supporting Information). By contrast, fat mass, often regarded as an energy storage tissue with low metabolic activity, negatively correlated with EE for all groups except the HFpEF+CL mice. In HFpEF+CL mice, fat mass appeared to have a divergent effect, which corresponded with increases in EE (Figure S5F, Supporting Information), suggesting that the fat mass of HFpEF+CL mice may have uniquely enhanced metabolic activity. Overall, these findings demonstrate that CL treatment significantly enhances systemic metabolic output, which is otherwise diminished in HFpEF. Additionally, in HFpEF+CL mice, increased EE is predominantly driven by lipid metabolism, as indicated by persistent metabolic inflexibility and reliance on FAO.

### 2.5 Adipose Tissue Mediates the Cardioprotective Effects of CL Treatment

**Figure 5:**
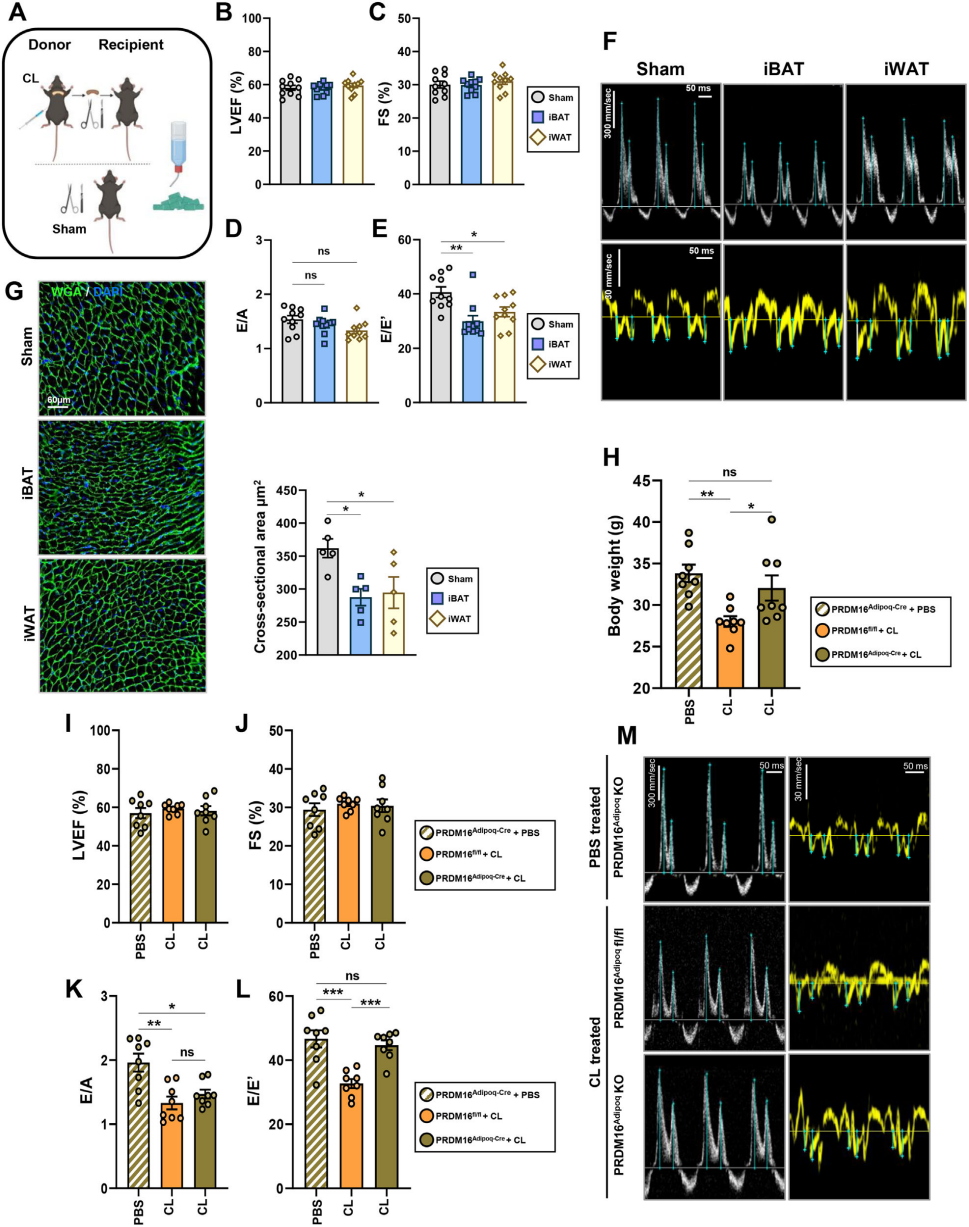
Thermogenic adipose tissue function is required for CL‐mediated cardioprotection. A) Schematic overview of the transplantation study design. B) Left ventricular ejection fraction and C) fractional shortening were measured from m‐mode traces. D,E) Parameters of diastolic function as determined by E/A and E/E’ recorded from apical four‐chamber views using Doppler modes; *n* = 10 mice per group. F) Representative images of pulsed‐wave and tissue Doppler cardiac cycles recorded using transthoracic echocardiography; scale bars indicate time (x‐axis) of 50 ms and velocity (y‐axis) of 300 mm sec^−1^. G) Representative images of WGA‐stained hearts and quantification of cardiomyocyte cross‐sectional area; *n* = 5 per group. Image magnifications are recorded using 40x objective; scale bars equal 60 µm. H) Body weights were recorded following HFpEF treatment in PRDM16^AdipoqCre^ KO mice and floxed controls. Echocardiographic measurements of I) left ventricular ejection fraction and J) fractional shortening recorded in PRDM16^Adipoq^ KO mice. Measurements of mitral K) E/A and L) E/E’ in PRDM16^Adipoq^ KO mice were recorded from the apical four‐chamber view; *n* = 8 mice per group. M) Representative images of pulsed‐wave and tissue Doppler cardiac cycles recorded using transthoracic echocardiography; scale bars indicate time (x‐axis) of 50 ms and velocity (y‐axis) of 300 mm sec^−1^. Data are presented as the mean ± SEM; significance values were determined by one‐way analysis of variance (ANOVA) followed by Tukey's multiple comparisons; *P ≤ 0.05, **P ≤ 0.01, ***P ≤ 0.001, ****P ≤ 0.0001.

To directly assess thermogenic AT as a potential mediator of CL‐mediated cardioprotection, we conducted a series of transplantation experiments involving CL‐treated donor mice and untreated wild‐type (WT) recipients. Approximately 100 mg of either iBAT or iWAT (taken from the peri‐lymph node region, which has the most abundant beige AT) was harvested from wild‐type donor mice on a chow diet, after they had received CL treatment. Donor tissues were then implanted into the visceral cavity, superior to the gWAT, into untreated WT recipient mice. After a one‐week recovery period, recipient mice were subjected to HFpEF treatment (**Figure** **5**A). Successful engraftment was confirmed at sacrifice based on the retention of brownish coloration in iBAT or iWAT transplants and vascularization of the grafted tissue (Figure S6A, Supporting Information). Following HFpEF treatment, body weight measurements indicated no significant differences between iBAT and iWAT recipients compared to sham‐operated mice (Figure S6B, Supporting Information). Echocardiographic assessment of systolic and diastolic function revealed that LVEF and FS remained similar across all groups (Figure 5B,C). When assessing diastolic parameters, a significant attenuation in the E/E’ of transplant recipients compared to sham controls was observed, indicating an improvement in diastolic function in transplant recipients (Figure 5D,F). Additionally, WGA staining revealed a notable reduction in cardiomyocyte hypertrophy in transplant recipients compared to sham‐operated mice (Figure 5G), mirroring the protective effects observed in HFpEF+CL‐treated mice (Figure 1L). These findings suggest that thermogenic AT transplantation recapitulates the cardioprotective effects of CL treatment, highlighting AT as a key mediator in preserving cardiac function under HFpEF conditions.

Next, to determine whether the thermogenic function of AT is required for CL‐mediated cardioprotection, we employed a loss‐of‐function genetic model. The *Adipoq‐*Cre; *Prdm16* ^fl/fl^ (PRDM16‐KO) ablates a crucial transcription factor, which blunts the induction of CL‐mediated AT thermogenesis, mostly in the iWAT. Moreover, AT isolated from PRDM16‐KO is also known to have reduced respiratory capacity.^[^ ^60^ ^]^ Knockout efficiency was validated at both gene and protein levels (Figure S6C,D, Supporting Information). The blunted induction of thermogenesis was shown by administering CL to floxed controls and PRDM16‐KO mice, followed by RT‐qPCR analysis of thermogenic markers in iBAT and iWAT. Thermogenic markers such as *Ucp1* and *Pgc1α*, as well as enzymes involved in DNL, including *Acly* and *Acc2*, were upregulated in both iBAT and iWAT with CL treatment, but in PRDM16‐KO mice, this response was significantly blunted, especially in the iWAT (Figure S6E,F, Supporting Information), confirming their diminished thermogenic capacity. To determine whether CL‐mediated cardioprotection required functional thermogenic capabilities in the AT, we treated floxed controls and PRDM16‐KO mice with either PBS or CL, followed by HFpEF induction. PRDM16‐KO mice gained significantly more weight than floxed controls after HFpEF treatment (Figure 5H), indicating worsened metabolic dysfunction. Echocardiographic analysis showed no significant differences in LVEF or FS between PRDM16‐KO and floxed controls (Figure 5I,J). However, diastolic function was notably impaired in PRDM16‐KO mice, with a significant increase in mitral E/E' values compared to floxed controls (Figure 5K–M). While CL provided cardioprotection to floxed controls, PRDM16‐KO mice showed no benefit, demonstrating that intact thermogenic function in AT is required for CL‐mediated cardioprotection. These findings highlight thermogenic AT as a crucial mediator in this HFpEF model, reinforcing its role in preserving cardiac function.

### 2.6 Genetic Expansion of Beige Adipose Tissue Provides Comparable Protection Against HFpEF

**Figure 6:**
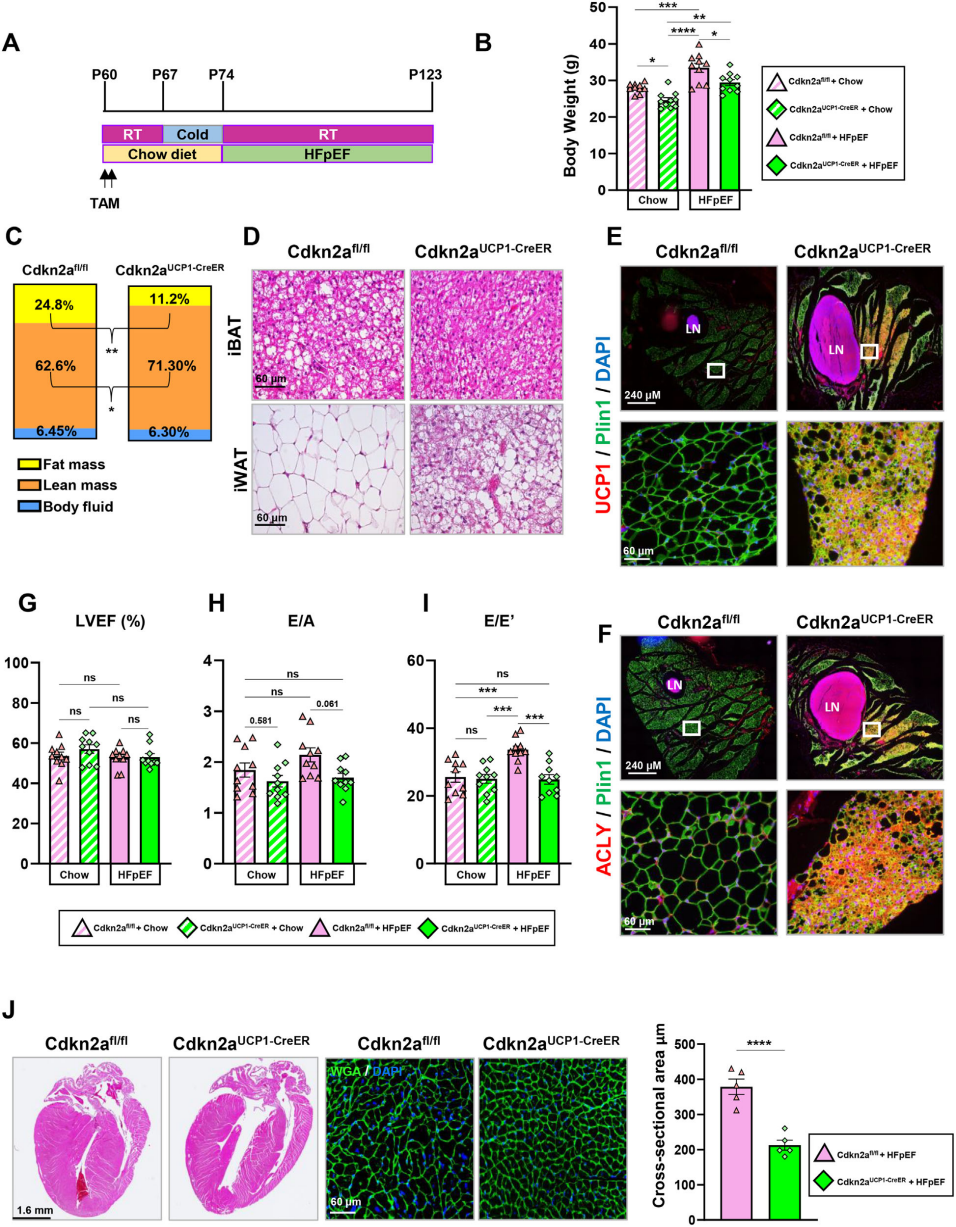
Genetic expansion of thermogenic adipose tissue confers cardioprotection against HFpEF. A) Treatment timeline for Cdkn2a^UCP1‐CreER^ KO mice exposed to HFpEF conditions. B) Net weight gained by Cdkn2a^UCP1‐CreER^ KO or floxed control mice exposed to either HFpEF treatment or chow diet; *n* = 10 per group. C) Body mass compositions were determined by nuclear magnetic resonance; *n* = 10 per group. D) Representative images adipose tissues from iBAT or iWAT stained with H&E. E,F) Immunofluorescence staining of beige adipose tissue regions located within the iWAT recorded in the lymph node region was performed to evaluate the expression of UCP1 and ACLY in Cdkn2a^UCP1‐CreER^ KO or floxed controls; image magnifications were recorded using a 40x or 5x objective; scale bars equal 60 or 240 µm; as indicated by labeling in figure; *n* = 5 per group. G) Transthoracic echocardiographic measurements of left ventricular ejection fraction. Pulsed wave and tissue Doppler modes used in the apical four‐chamber measurements recorded the mitral H) E/A and I) E/E’; *n* = 10 mice per group. J) H&E‐stained hearts sectioned in the longitudinal plane and WGA‐stained hearts sectioned in the transverse plane show cardiomyocyte hypertrophy and quantification of cross‐sectional myocyte area; *n* = 5 per group. Data are presented as the mean ± SEM; where only two groups were compared significance values are determined by two‐tailed unpaired Student's *t*‐test, where more than two groups were compared to each other significance values were determined by one‐way analysis of variance (ANOVA) followed by Tukey's multiple comparisons; *P ≤ 0.05, **P ≤ 0.01, ***P ≤ 0.001, ****P ≤ 0.0001.

To further investigate the role of thermogenic AT in HFpEF, we employed a gain‐of‐function genetic model, Ucp1‐Cre^ERT2^; Cdkn2a^fl/fl^ (Cdkn2a^UCP1‐CreER^ KO), which promotes thermogenic AT expansion by deleting *Cdkn2a* in UCP1‐expressing cells.^[^ ^40^, ^61^ ^]^ Additionally, this model extends the lifespan of newly formed beige adipocytes following thermogenic stimulation, leading to sustained EE enhancements in the context of overnutrition.^[^ ^40^, ^61^ ^]^ Unlike prior models (CL treatment or AT transplantation), we can specifically target UCP1+ proliferative beige adipocytes and test their contributions to HFpEF outcomes here. To assess this, control and Cdkn2a^UCP1‐CreER^ KO mice were cold‐exposed to induce beige AT expansion and subsequently placed on HFpEF treatment or a chow diet (**Figure** **6**A). As expected, HFpEF treatment resulted in significant weight gain in both control and KO mice; however, net weight gain in KO mice appeared to be attenuated (Figure 6B). We next evaluated how KO mice differed from control mice following HFpEF treatment. NMR measurements revealed that KO mice had reduced fat mass and increased lean mass compared to the floxed controls (Figure 6C; Figure S7A, Supporting Information). This corresponded with reductions in recorded weights of white AT, but not brown AT (Figure S7B, Supporting Information), reflecting the specificity of increased metabolic activity due to the expansion of new beige adipocytes residing within white AT depots. Other organ weights were unaffected except for a significant reduction in heart weight (Figure S7C, Supporting Information).

We next sought to evaluate systemic indicators of metabolic health. Results from a glucose tolerance test indicated that glucose sensitivity was not affected (Figure S7D, Supporting Information). Histological analysis of AT depots showed a stark difference between KO mice and controls following HFpEF treatment. In KO mice, beige AT was readily observable in the iWAT, as indicated by the abundance of dense multilocular adipocytes in iWAT. Likewise, in the iBAT, lipid infiltration was more pronounced in controls than in KO mice after HFpEF treatment (Figure 6D; Figure S7E, Supporting Information). Previously, we recognized DNL as a relevant metabolic feature of chronic thermogenesis affected by HFpEF and CL treatment, whereas lipolysis appeared to be unaffected. Therefore, we sought to compare the expression of UCP1, along with the DNL marker ACLY, in the iWAT KO and control mice after HFpEF treatment. Immunofluorescent staining revealed a tandem upregulation of UCP1 and ACLY in the iWAT of Cdkn2a^UCP1‐CreER^ KO (Figure 6E,F), reinforcing the link between DNL and sustained thermogenesis.

To assess the impact of this thermogenic phenotype on cardiac function, we performed echocardiography. Measurements of LVEF (Figure 6G) and FS (data not shown) indicated that systolic function remained comparable between the KO and control groups. Diastolic parameters indicated that beige AT expansion mediated cardioprotective effects. A trend of reduced E/A values was observed in Cdkn2a^UCP1‐CreER^ KO mice, both in the chow diet and HFpEF‐treated group (Figure 6H). As expected, floxed controls on HFpEF treatment exhibited a significant elevation of the E/E' ratio, indicative of diastolic dysfunction. This effect, however, appeared to be significantly attenuated in KO mice with HFpEF (Figure 6I), indicating that beige adipose expansion mediates cardioprotection. Histological analysis further demonstrated reduced hypertrophic remodeling and a significant decrease in cardiomyocyte hypertrophy, as measured by the cross‐sectional area of WGA‐stained hearts (Figure 6J), consistent with the previously observed cardioprotective effects of thermogenic AT. These results highlighted the beneficial role of beige AT expansion in preserving cardiac structure and function in obesity‐related HFpEF. Additionally, genetically targeting UCP1+ thermogenic proliferative beige adipocytes emerges as a key cellular target with substantial potential for improving cardiac outcomes in this form of HFpEF.

### 2.7 Thermogenic Adipose Tissue Alters the Cardiac Lipidome

Unlike its role in the liver, where DNL appears harmful in the context of obesity, DNL may have beneficial roles in the AT during obesity^[^ ^62^ ^]^ and may support the synthesis of specific lipids capable of regulating metabolism,^[^ ^63^ ^]^ inflammation,^[^ ^64^ ^]^ and cardiac function,^[^ ^65^, ^66^, ^67^ ^]^ while also providing a substrate for oxidation during chronic thermogenesis.^[^ ^68^ ^]^ The observed association between enhanced expression of DNL enzymes and cardioprotection in both CL‐treated mice and Cdkn2a^UCP1‐CreER^ KO prompted us to consider how lipid metabolism in the AT may be linked to the heart. In the acute period following the induction of thermogenesis, AT are known to coordinate systemic metabolic activities by releasing NEFA to be converted to BHB in the liver. While this particular form of crosstalk remains well recognized, the ways in which other distal tissues respond to the induction of thermogenesis remain poorly characterized. To assess the potential role which AT may have in governing cardiac lipid composition, we treated chow‐fed wild‐type mice with CL and assessed their hearts at 6‐ and 24‐hours post CL‐treatment using Oil Red O (ORO) staining to visualize lipid deposition. At 6 hours post‐CL treatment, there was a dramatic increase in lipid content. Remarkably, by the 24‐hour time point, lipid content had recovered to baseline levels (Figure S8A, Supporting Information). These findings describe how the induction of AT thermogenesis affects myocardial lipid content. To address whether CL treatment had sustained effects on myocardial lipid content, we compared ORO staining in HFpEF and HFpEF+CL hearts. However, no differences occurred between these two groups as both appeared to have similar increases in lipid deposition compared to CTRL mice (Figure S8B, Supporting Information).

**Figure 7:**
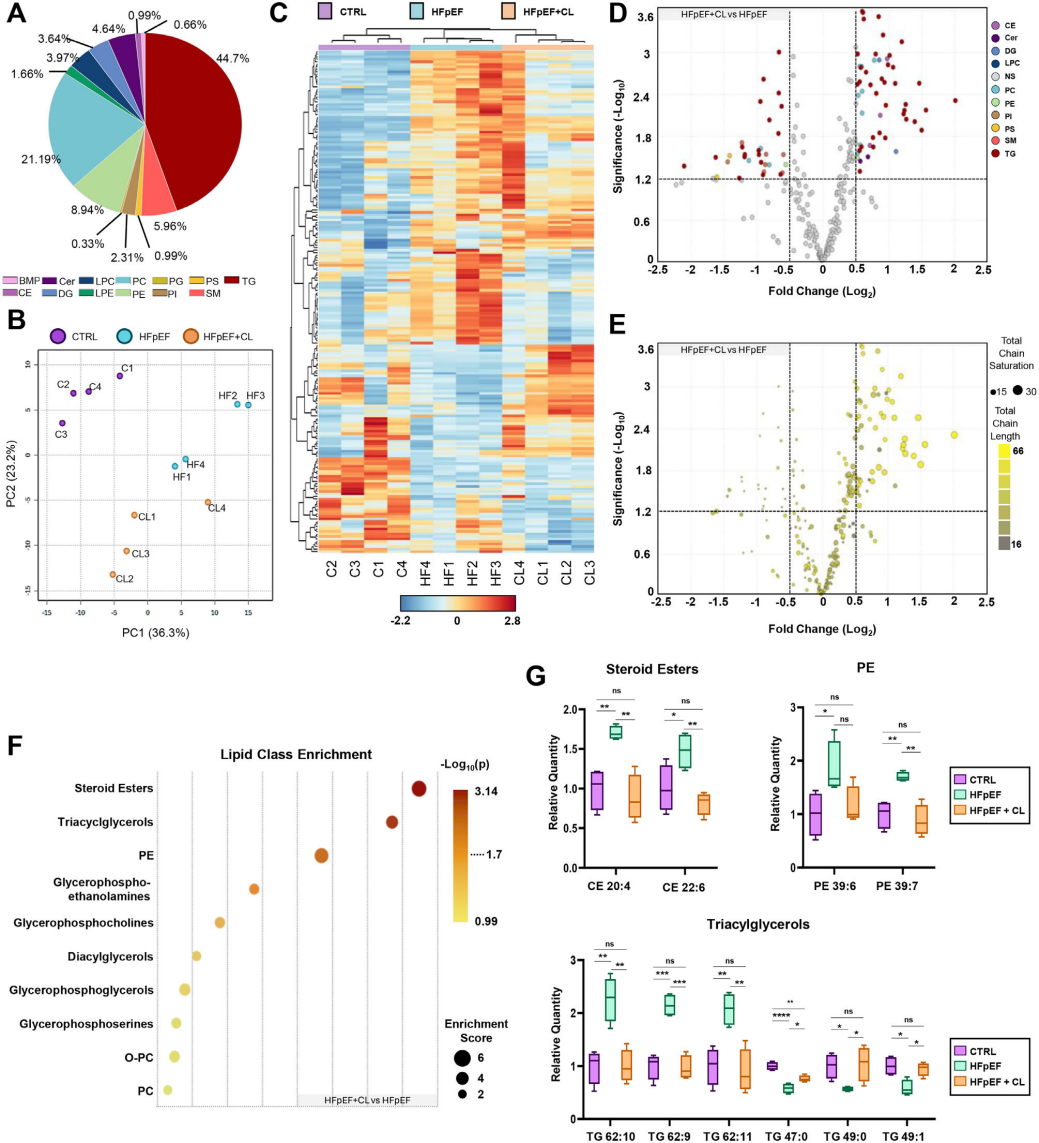
Thermogenic conditioning of adipose tissue promotes alterations in the cardiac lipidome following HFpEF treatment. A) A survey of all lipid classes identified by LC‐MS/MS reported the relative proportion displayed in a pie chart. B) 2D principal component analysis displaying the amount of variation observed between the indicated treatment groups. Hierarchical clustering results were performed on rows and columns. Each row contains an expression value for an individual lipid species. C) Dendrogram linkages on top and left of the heatmap describe the relationship between lipid species or samples that corresponded to the treatment groups indicated by the color displayed above. D) Volcano plots display lipid species that experienced significant differences in relative abundance between the two HFpEF and HFpEF+CL (Log_2_FC > ± 0.5, *p* < 0.05), colored according to the indicated lipid class, or E) displaying the total chain saturation level as indicated by size and chain length as indicated by color. F) Results obtained from quantitative enrichment analysis show the most significantly affected lipid classes that differentiate HFpEF+CL and HFpEF. G) Box plots of individual lipids from the three most significant classes identified by enrichment analysis show the relative quantity of lipids measured across all three treatment groups. Significance values were determined by one‐way analysis of variance (ANOVA) followed by Tukey's multiple comparisons; *P ≤ 0.05, **P ≤ 0.01, ***P ≤ 0.001, ****P ≤ 0.0001.

To resolve potential compositional differences not recognized by histological approaches, we employed positive ion LC‐MS/MS to profile the cardiac lipidome to characterize over 300 nonpolar lipid species across 13 lipid classes (**Figure** **7**A). Principal component analysis (PCA) revealed a clear distinction between CTRL, HFpEF, and HFpEF+CL mice (Figure 7B), indicating the CL treatment promoted significant changes in myocardial lipid remodeling. Hierarchical clustering of differentially regulated lipids showed that HFpEF treatment increased the quantity of a broader range of different species than that which occurred in HFpEF+CL mice, while each respective group appears to have unique clusters of enriched lipids. To better recognize compositional differences between the cardio‐protected state (HFpEF+CL) and disease state (HFpEF), volcano plots of differentially regulated lipids (Log_2_FC > ± 0.5, *p* < 0.05) were constructed to display how the lipid class and lipid features (chain length and saturation level) were affected (Figure 7D,E). Analysis of chain length and saturation levels revealed that HFpEF hearts were enriched in lipids with longer chain lengths and higher degrees of saturation, while HFpEF+CL hearts were, by contrast, enriched with lipids with shorter chain lengths and lower degrees of saturation (Figure 7E). These findings align with what is known about lipid stress in the heart, as long‐chain fatty acids mediate lipotoxic stress, whereas short‐chain fatty acids have anti‐inflammatory roles.^[^ ^69^ ^]^ In performing a quantitative enrichment analysis, steroid esters, triacylglycerols, and phosphatidylethanolamine (PE) emerged as the most significantly affected class of lipids, distinguishing HFpEF from HFpEF+CL (Figure 7F). Box plots of selected lipids from each of these categories show individual lipids of interest from each of these classes that appear to be significantly affected by HFpEF treatment, which are normalized in HFpEF+CL mice (Figure 7G). HFpEF was also compared directly to CTRL hearts in order to showcase the full extent of lipid remodeling that occurs under pathological conditions. Volcano plots of differentially regulated lipids featuring lipid class and lipid features (Figure S9A,B, Supporting Information) showed that the increased lipid deposition resulting from HFpEF treatment affected a broad range of different lipid classes and chain features, appearing nonspecific. Quantitative enrichment analysis indicated that phosphatidylcholines (PC) and glycerophosphoethanolamines were among the most significantly affected classes of lipids in HFpEF compared to CTRL (Figure S8C, Supporting Information).

Overall, these findings showcase the ability of AT to mediate dynamic alterations in myocardial lipid content during thermogenesis and reveal how the targeting of AT with CL mediates significant compositional differences in the cardiac lipidome after HFpEF treatment. Lipid chain analysis described how CL treatment promoted changes in the cardiac lipidome that appeared to be beneficial, consisting of shorter‐chain, less saturated lipids as compared to HFpEF hearts, where longer‐chain, highly saturated lipids appeared to be more prevalent.

## 3 Discussion

In this study, we utilized an obesity‐related two‐hit heart failure with preserved ejection fraction (HFpEF) model to explore the therapeutic potential of directly targeting adipose tissue (AT). Our findings demonstrate that AT acts as a direct mediator of cardiac dysfunction and that targeting of the AT effectively mitigates disease progression, resulting in improved diastolic function and attenuated hypertrophic remodeling. This work introduces a conceptual shift by positioning AT as a primary therapeutic target in obesity‐related HFpEF. Furthermore, using a gain‐of‐function mouse model, we highlight beige adipocytes as potential candidates for targeted cellular therapies. Additionally, we identified how the targeting of AT leads to changes in the cardiac lipidome, offering new insights into metabolic aspects of adipose‐heart crosstalk.

Although the use of β3‐AR agonist was well‐suited for our studies, given that murine cardiomyocytes appear unresponsive to CL‐316243 (CL),^[^ ^46^ ^]^ translational considerations must acknowledge the potential adverse effects β3‐AR stimulation may have on failing human hearts.^[^ ^70^ ^]^ However, a variety of other agents, including natural compounds,^[^ ^71^ ^]^ metabolites, and lipokines^[^ ^63^, ^72^ ^]^ may be better suited for use in humans. Interestingly, newly marketed diabetes drugs, specifically sodium‐glucose cotransporter‐2 inhibitors (SGLT2i) and glucagon‐like peptide‐1 (GLP‐1) agonists,^[^ ^6^, ^7^ ^]^ appear beneficial in HFpEF and have previously described thermogenic properties.^[^ ^73^, ^74^ ^]^ Conceptually, this aligns with our results and supports the notion that AT modulation may contribute to their mechanism of benefit in HFpEF. Our use of the Cdkn2a^UCP1‐CreER^ KO model further highlighted a specific cellular precursor within inguinal white AT (iWAT), suggesting a unique opportunity to develop precise targeted cell‐based therapies as an alternative to traditional pharmacological approaches.

Obesity and aging, key drivers of HFpEF, are associated with metabolic and AT dysfunction, including loss of thermogenic capacity^[^ ^31^, ^34^, ^75^ ^]^ and reduced energy expenditure (EE).^[^ ^76^, ^77^ ^]^ Preconditioning AT with CL appeared to promote a healthier expansion of the AT, preserved interscapular brown AT (iBAT) integrity, and enhanced EE. Chromatin remodeling induced by thermogenesis may be sustained for relatively long periods of time, shaping how the AT responds to future challenges.^[^ ^78^ ^]^ However, we believe the protective effects associated with healthier AT expansion observed in our study would likely weaken over time, given the continuous nature of the metabolic stress presented by HFpEF treatment. Our experimental timeline was therefore well‐suited to capture the protective effects over a meaningful period. The systemic benefits of targeting the AT correlated with attenuated diastolic dysfunction, reduced hypertrophic remodeling, and significant changes in the cardiac lipidome, which appear beneficial.^[^ ^69^ ^]^ Recent studies recognize the importance of cardiac lipids in providing cardiomyocyte membrane structure, regulating signaling events, and mediating transmembrane transportation. Moreover, dynamic remodeling of the cardiac lipidome is known to occur during the development of heart failure and aging.^[^ ^79^, ^80^, ^81^, ^82^ ^]^ Conversely, lifestyle interventions such as exercise, which have beneficial effects for HFpEF patients,^[^ ^83^ ^]^ are also known to induce dynamic remodeling of the myocardial lipidome,^[^ ^84^ ^]^ emphasizing how the cardiac lipidome is a dynamic feature of the heart that changes during both health and disease.

While both iBAT and iWAT mediated cardioprotection in the transplantation experiments, the importance of the iWAT was more clearly supported by our experimental data overall. This may reflect a complex inter‐depot coordinated response or suggest the involvement of multiple mechanisms of cardioprotection.^[^ ^54^, ^85^ ^]^ However, in the PRDM16‐KO model, the response to CL treatment was more severely blunted in the iWAT. Likewise, with the Cdkn2a^UCP1‐CreER^ KO model, the effects occur mostly in the iWAT, where proliferation of beige adipocytes occurs following a thermogenic stimulus.^[^ ^40^ ^]^ These experimental observations, alongside clinical evidence, underscore how specific quality and characteristics of AT, rather than merely total mass, are central to the pathophysiology of obesity‐related forms of HFpEF. In humans, for example, regional differences in AT expansion (subcutaneous vs visceral) appear linked to key functional parameters in HFpEF.^[^ ^9^, ^10^, ^11^, ^12^, ^13^ ^]^ Likewise, the waist‐to‐hip ratio, which recognizes differences in adipose distribution, has been shown to outperform BMI in predicting mortality.^[^ ^86^ ^]^ Monitoring specific AT features may therefore be useful in developing a better understanding of how specific features of adiposity relate to subtypes in HFpEF and help pave the way for more personalized approaches.^[^ ^87^ ^]^ Similarly, the implications of experimental findings relating the AT and HFpEF should recognize the importance of using well‐defined models that are tailored to specific features or subgroups within the HFpEF population. For this reason, we have made use of only age‐matched male mice, excluding variables like female sex and aging, which are known to affect thermogenic capacity^[^ ^35^ ^]^ and feature in related forms of HFpEF.^[^ ^16^ ^]^ Although thermogenic AT in humans associates with beneficial outcomes in the context of cardiometabolic diseases,^[^ ^30^ ^]^ Experimental approaches to define the nature of this relationship have remained challenging as both positive and negative effects have been attributed to this activity in other forms of cardiovascular disease.^[^ ^39^, ^88^, ^89^, ^90^, ^91^ ^]^ This may reflect how outcomes are shaped by different models, the duration of thermogenic activation, or the ways in which particular aspects of thermogenic activity or cardiovascular health are defined and measured. Thus, while adipose dysfunction is likely involved in other forms of HFpEF, it remains important to consider the context and limitations of experimental evidence to faithfully determine what the implications may be for highly heterogeneous conditions, such as HFpEF. Moreover, our own studies remain limited in their ability to address the role of AT even in this model since we have only evaluated a limited number of AT depots. Although the depots that we evaluated remain among the most studied, it should be noted that other depots, which have potentially relevant roles in the context of HFpEF, such as perivascular or epicardial AT depots, were not evaluated in our studies.

In summary, we demonstrate that AT serves as a determinant of cardiovascular outcomes in obesity‐related HFpEF. Pre‐treatment with CL appears to promote healthier forms of AT expansion, which resist the effects of HFpEF and enhance systemic metabolic function. These AT‐mediated effects improve cardiac structure and function and facilitate lasting changes in the cardiac lipidome. Given the high degree of heterogeneity among HFpEF patient populations, these findings underscore the need for tailored experimental models to fully elucidate the specific roles of AT dysfunction across different HFpEF subgroups.

## 4 Experimental Section

### Animal Experiments

All animal experiments followed instructional guidelines with attention to required ethical regulations. Male C57/Bl6 mice aged 8–10 weeks were selected for the treatment groups. Mice were maintained on a 12‐hour light/dark cycle and housed at ambient temperatures. A two‐hit model of HFpEF was established by treatment with high fat diet (HFD) (D12492, Research Diets) and water containing *N* ^ω^‐nitro‐L‐arginine methyl ester (L‐NAME) (0.5 g L^−1^), buffered to pH 7.4, changed every 48 hours. HFpEF treatments were maintained for 7‐week periods. Control diets were fed standard chow (Teklad) and regular drinking water. CL316243 was purchased from Tocris Bioscience and dissolved in sterile water. CL treatment consisted of (1.0 mg kg^−1^ body weight) daily intraperitoneal (IP) injections for seven consecutive days. PRDM16^fl/fl^ (#032160) and Adipoq‐Cre (#028020) mice were purchased from JAX. Ucp1‐Cre^ERT2^ and Cdkn2a^fl/fl^ mice were generously provided by Dr. Eric N. Olson (University of Texas Southwestern Medical Center). Cre recombination was induced by administering tamoxifen (Cayman Chemical) 1.5 mg kg^−1^ body weight dissolved in sunflower oil (Sigma) through IP injection for two consecutive days. All animal experiments were approved by the Animal Care and Use Committee (ACC#22‐186) of the University of Illinois Chicago (UIC). All experiments were performed in accordance with the relevant UIC guidelines and regulations administered through the Office of Animal Care and Institutional Biosafety (OACIB).

### Transthoracic Echocardiography

Transthoracic echocardiography was performed on unconscious mice. Anesthesia was induced using 3% isoflurane supplied to an induction chamber, whereafter, the lack of hind paw response was used to confirm induction. Mice were then secured to a warming table with ECG electrodes to monitor heart rate. Depilatory cream was used to remove hair. Concentrations of isoflurane were maintained between 0.5 and 1.5% to maintain a target heart rate of 450 ± 50 BPM. Ultrasound scans were obtained with the Vevo2100 imaging system (Visual Sonics Inc, Toronto, ON, Canada) with the MS550D probe using a center frequency of 40 MHz. M‐mode tracings were measured from the short‐axis view. Mitral inflow and tissue velocities were measured using pulsed wave Doppler and tissue Doppler modes from the apical four‐chamber view. Mice were carefully monitored following anesthesia to ensure complete recovery. All measurements were obtained from at least three consecutive and consistent cardiac cycles.

### Tail‐Cuff Blood Pressure Monitoring

Blood pressure readings were measured using the CODA tail cuff monitoring system (KentScientific). Mice were first acclimated to the instrument for three consecutive days. Before recording measurements, mice were briefly anesthetized with isoflurane in an induction chamber with 2.5% isoflurane, then restrained, and placed on the warming table. Tail temperatures were monitored and maintained between 32 and 35 °C. Data were recorded using the default preferences with five acclimation cycles and ten data collection cycles. Mean arterial pressures were calculated as (SBP+2(DBP))/3.

### Intraperitoneal Glucose Tolerance Test

Mice were fasted for five hours during the day and then administered a bolus of high glucose solution (125 mg mL^−1^) via intraperitoneal injection (1.25 mg g^−1^ body weight). Tail blood was analyzed for glucose levels (mg dL^−1^) and measured using the Contour next EZ (Bayer) blood glucose monitoring system. Blood glucose levels were measured before glucose administration (0 minutes) and then at the following intervals after glucose administration: 15, 30, 45, 60, 90, and 120 minutes.

### Serum Collection

Blood lancets (Medipoint) were used to make a retro‐orbital puncture, and blood was collected using capillary serum collection tubes (SAI, MVC‐S) in fed mice. Samples were incubated at room temperature for 30 minutes and then centrifuged at 1500 x G for 10 minutes in a refrigerated centrifuge. NEFA was measured using colorimetric enzymatic assays (Wako Diagnostics USA) by the Metabolic Phenotyping Core at UT Southwestern.

### Lipid Profiling

Extracted hearts were immediately flash‐frozen in liquid nitrogen and then homogenized in ice‐cold HPLC‐grade isopropanol >10 µL mg^−1^ tissue. Homogenates were then vortexed for a total of 10 minutes and centrifuged in a 4‐degree centrifuge at 14 000 RPM for 10 minutes. The supernatant was then collected and transferred to a clean centrifuge tube and stored in a minus‐80 freezer. Positive ion LC‐MS/MS was performed by the Proteomics and Metabolomics Core Facility at Weill Cornell Medicine. Intensity values for lipid profiling provided relative quantification of the same lipid species across different samples, but not of different lipid species. Analysis was performed using the MetaboAnalyst 6.0.^[^ ^92^ ^]^ Variables with >50% missing values were excluded from analysis and auto‐scaled using mean normalized values. Dot plots and volcano plots were constructed using Scimago Graphic.

### Histology

Hearts or adipose tissue depots were fixed in 10% formalin. Hearts were fixed in formalin overnight, along with interscapular brown tissues, while inguinal and perigonadal depots were fixed for an additional 48 hours. Tissue was then processed using an automated tissue processor for paraffin embedding. Cardiac sections were cut into 7 µm slices, while adipose tissue sections were cut into 5 µm slices. Paraffin sections were incubated at 45 °C overnight. Following deparaffinization, slides were incubated in hot citrate buffer (Sigma, C9999) for antigen retrieval. WGA (Invitrogen, W11261) and Isolectin (Invitrogen I21412) were used to determine cardiomyocyte size and vascular density. Picrosirius red stains were prepared by dissolving 0.1 g of Direct Red 80 (Millipore, 365548) into 100 mL of saturated picric acid and then washing with a 0.5% acetic acid solution in DI water. ImageJ ROI tool was used to trace and record cardiomyocyte areas after calibrating the image size to the scale bar. Percentage area measurements for vascular density and fibrosis area were performed by creating RGB stacks for binary images to measure pixels.

For IHC/IF, sections were washed in 3% H_2_O_2_ for 30 minutes after deparaffinization and then blocked in 10% goat serum before overnight incubation with a primary in a 4 °C cold room. After washing, sections were incubated in an anti‐biotin secondary antibody (Jackson ImmunoResearch, 111‐065‐144, 1:1000) for two hours, followed by three washes in PBS. Detection using an ABC Peroxidase kit (Thermo, 32020) and Pierce DAB substrate (Thermo, 34002) was carried out according to the manufacturer's specifications. After development, slides were stained with hematoxylin and mounted with cytosol (Fischer, 8310‐16). Images were recorded using Lecia DMi8 with Leica LAS Application Suite X 3.7.4 software.

For Oil Red O stains, fresh hearts are extracted and immediately frozen with O.C.T. compound. Frozen sections were airdried for one hour, briefly rinsed in water, and dipped in 60% isopropyl alcohol before incubating in an Oil Red O staining solution for 10 minutes (Thermo, AAA1298914). Stock Oil Red O (0.3 g of Oil Red O in 100 mL Isopropanol) was diluted 3:2 in H_2_0 and then filtered to make the staining solution. After incubation in a staining solution, slides were dipped in 60% isopropyl alcohol, rinsed in H_2_0, and then mounted with aqueous mounting media.

### Cardiac Cellular Isolation

Dynabeads were prepared the night prior to EC isolation by mixing (2.5 µg) of ICAM‐2 per 2 × 10^7^ beads on an end‐over‐end rotator overnight at 4 °C. Hearts were then isolated and minced into small pieces and incubated with enzyme solution (Collagenase II; 0.2% in serum‐free DMEM) for 50 minutes at 37 °C with gentle shaking. The suspension of a 40 µm disposable cell strainer into a 50 mL tube pre‐filled with neutralization media (FBS‐containing PBS) and then centrifuged at 1200 rpm for 8 minutes at 4 °C, and resuspended in the bead washing solution and incubated with ICAM‐2 coated Dynabeads for 20 minutes at room temperature with shaking. The bead suspension was then placed on a magnetic rack and washed four times with bead washing solution and once with PBS, and then lysed with RIPA+ protease inhibitor. A Langendorff apparatus was used to isolate cardiomyocytes. Mice were first anesthetized, and then the hearts with intact aorta were harvested. After cannulating the aorta, hearts were perfused with the warmed isolation buffer (130 mm NaCl, 25 mm KCl, 10 mm KH_2_PO_4_, 2 mm MgSO_4_, 10 mm glucose, 20 mm taurine, 5 mm creatine, 100 mm potassium glutamate, 10 mm aspartic acid, 10 mm HEPES, 50 µm CaCl_2_) and then briefly subjected to further digestion (Type 2 Collagenase). The heart was removed from the Langendorff apparatus and placed in a dissociation buffer (10% FBS and 25 µm Blebbistatin in KB buffer). After removing the atria and right ventricle, left ventricular myocytes were dissociated into solution. Calcium was adjusted prior to adding culture media (consisting of MEM supplemented with 25 mm HEPES, 10% FBS, and penicillin/streptomycin). Cardiomyocyte lysates were obtained using RIPA buffer supplemented with protease and phosphatase inhibitors.

### RNA Isolation and qPCR

Tissues were excised, flash frozen in liquid nitrogen, and then stored in a −80 °C freezer. TRIzol was then added to frozen tissues, which were allowed to briefly thaw on ice. Tissues were homogenized using TissueRuptor (Qiagen), centrifuged at 1500xg for two minutes to remove debris, and then isolated using a Direct‐zol RNA isolation kit (Zymogen). An additional two rounds of centrifugation for adipose tissues were included to remove lipid contamination, followed by chloroform extraction. RNA concentrations were measured using a BioDrop. cDNA reactions were prepared using one µg of RNA with MultiScribe Reverse Transcriptase, followed by qPCR using SYBR green reagents and a QuantStudio 7 Flex Real‐Time PCR system.

### Protein Isolation and Western Blotting

Tissues were excised, flash frozen in liquid nitrogen, and then stored in a −80 °C freezer. RIPA buffer with added Halt protease cocktail inhibitor was then added to frozen tissues, which were allowed to thaw on ice briefly. Tissues were homogenized using TissueRuptor (Qiagen) and then centrifuged at 4 °C at 1500xg for 15 minutes to remove debris. For adipose tissues, an additional two rounds of centrifugation were performed. The supernatant was then carefully collected to avoid the fat cake, which was separated to prevent lipid contamination. Protein concentrations were then measured using the Pierce Rapid Gold BCA kit. Western blot membranes were blocked in SuperBlock blocking buffer for one hour, incubated in primary antibodies overnight in a 4 °C cold room, washed, and then incubated with secondary antibodies for two hours, and imaged using an iBright Imaging System (Thermo). When applicable, membranes were stripped with Restore PLUS (Thermo) stripping buffer.

### Indirect Calorimetry

Mice were housed individually and acclimatized to the metabolic chambers (Promeethion System, Sable System International) at the UIC Biologic Resources Laboratory for 2 days before data collection was initiated. For the subsequent 3 days, food intake, VO_2_, VCO_2_, energy expenditure, and physical activity were monitored over a 12 h light/dark cycle with food provided ad libitum. For energy expenditure analysis, raw data were analyzed using the web server tool CalR.^[^ ^93^ ^]^ For body composition analysis, the total fat and lean mass were assessed with Bruker Minispec 10 whole body composition analyzer (Bruker).

### Flow Cytometry

Sample collection was performed at consistent times for each group. Peripheral blood was obtained via lateral facial vein puncture. Mice were euthanized and perfused via the left ventricle with ice‐cold phosphate‐buffered saline (PBS) to remove circulating blood. Adipose tissues were collected and briefly minced with scissors and then placed into a conical vial on ice with digest buffer (1xHBSS, 3%BSA, Collagenase II [288 U mL^−1^], CaCl_2_[1 m], MgCl_2_[1 m], ZnCl_2_[0.1 m], ddH_2_0). Samples were briefly vortexed to promote dissociation, and then incubated at 37 °C for 15 minutes on a shaker, and then briefly vortexed once again and incubated for another 15 minutes. Digested cells were then centrifuged at 200xg for 5 minutes at 4 °C, resuspended in RPMI+10% FBS, and centrifuged at 200xg. Pellets were then resuspended in ACK lysis buffer and incubated for 2 minutes for RBC lysis, then quenched with RMPI+10% FBS. The suspension was then filtered through a 40 µm filter placed in a clean conical vial and then centrifuged at 200xg for 5 minutes at 4 °C. Pellets were then resuspended in FACS buffers (EDTA [10 mm], 0.5% BSA, PBS) and labeled with the indicated fluorophore‐conjugated antibodies and analyzed on a BD FACSCelesta flow cytometer (BD Bioscience) with FACSDIVA software (BD Biosciences). Data were analyzed using FlowJo software (FlowJo, LLC).

### Transplantation Studies

All surgical procedures were performed in compliance with the ACC‐approved protocol. Mice were administered meloxicam (5 mg kg^−1^ daily I.P. injections for 2 days) as an analgesic. Anesthesia was induced as described above. A warming platform was used to avoid heat loss during surgery. Adipose tissue depots from donor mice were excised and briefly washed in a warm sterile saline solution before being transferred to recipient mice. Prior to incision, surgical sites were cleaned in Betadine, and then abdominal cavities were exposed with sterilized surgical tools. Recipient mice were given ≈100 mg of iBAT or iWAT from CL‐treated donors and carefully placed within the fold of the gWAT. For sham‐treated mice, gWAT tissue was exposed and returned to its original position. The body cavities were then sealed with surgical sutures. Mice were then carefully monitored following the procedure for any signs of infection, distress, or discomfort.

### Statistical Analyses

Statistical significance was determined using Prism software or Excel. The values are presented as the mean ± standard deviation (SD) or standard error of the mean (SEM) as indicated by the figure legends. Statistical differences were determined by a 2‐tailed, unpaired Student's *t*‐test or one‐way ANOVA with Tukey's multiple comparison test as appropriate. Values of *p* < 0.05 were considered statistically significant. Statistical analysis of lipidomic data was performed using https://www.metaboanalyst.ca/ and graphed using scimago graphica. The supplemental appendix contains a list of materials and reagents used.

## Conflict of Interest

The authors declare no conflict of interest.

## Author Contributions

J.J., Y.J., and S.‐G.O. conceptualized the idea for the study. J.J., Z.H., J.P., G.Y., S.Z., J.K., Y.K., and S.B.N. designed the methodology and performed the investigation. C.W.L., W.H.L., S.‐B.O., Y.J., and S.‐G.O. performed data interpretation and discussion. S.P., Y.J., and S.‐G.O. performed the supervision. J.J., Y.J., and S.‐G.O. wrote the manuscript.

## Supporting information



Supporting Information

## Data Availability

The data that support the findings of this study are available from the corresponding author upon reasonable request.
