# Supplementary material for: Targeting Adipose Tissue Function Protects Against Heart Failure with Preserved Ejection Fraction
Source: Adv Sci (Weinh). 2025 Nov 23;13(7):e06106. doi: 10.1002/advs.202506106 (PMC12866728; doi:10.1002/advs.202506106)
Supplement: Supplementary file 1 — Supporting Information [file ADVS-13-e06106-s001.docx]

**SUPPLEMENTAL APPENDIX**

**Targeting Adipose Tissue Function Protects Against Heart Failure with Preserved Ejection Fraction**

Jordan Jousma PhD^1^, Zhenbo Han PhD^1^, Jooman Park PhD^2^, Gege Yan PhD^1^, Sen Zhang PhD^1^, Youjeong Kwon MSc^1^, Sarath Babu Nukala PhD^1^, Jindpreet Kandola MSc^1^, Sandra Pinho PhD^1^, Chong Wee Liew PhD^2^, Yuwei Jiang PhD^2,4,5*^, Sang-Ging Ong PhD^1,6*^

**METHODS**

**EXPERIMENTAL ANIMALS**

All animal experiments followed instructional guidelines with attention to required ethical regulations. Male C57/Bl6 mice aged 8-10 weeks were selected for the treatment groups. Mice were maintained on a 12-hour light/dark cycle and housed at ambient temperatures. A two-hit model of HFpEF was established by treatment with HFD (D12492, Research Diets) with water containing L-NAME (0.5 g L^-1^), buffered to pH 7.4, and changed every 48 hours. HFpEF treatments were maintained for 7-week periods. Control diets were fed standard chow (Teklad) and regular drinking water. CL316,243 was purchased from Tocris Bioscience and dissolved in sterile water. CL treatment consisted of (1.0 mg kg^-1^ body weight) daily intraperitoneal (IP) injections for seven consecutive days. PRDM16^fl/fl^ (#032160) and Adipoq-Cre (#028020) were purchased from JAX. Ucp1-Cre^ERT2^ and Cdkn2a^fl/fl^ mice were generously provided by Dr. Eric N. Olson (University of Texas Southwestern Medical Center). Cre recombination was induced by administering tamoxifen (Cayman Chemical) 1.5 mg kg^-1^ body weight dissolved in sunflower oil (Sigma) through IP injection for 2 consecutive days. All animal experiments were approved by the Animal Care and Use Committee of the University of Illinois Chicago (UIC). All experiments were performed in accordance with the relevant UIC guidelines and regulations administered through the Office of Animal Care and Institutional Biosafety (OACIB).

**TRANSTHORACIC ECHOCARDIOGRAPHY**

Transthoracic echocardiography was performed on unconscious mice. Anesthesia was induced using 3% isoflurane supplied to an induction chamber, whereafter, the lack of hind paw response was used to confirm induction. Mice were then secured to a warming table with ECG electrodes to monitor heart rate. Depilatory cream was used to remove hair. Concentrations of isoflurane were maintained between 0.5-1.5% to maintain a target heart rate of 450 ± 50 BPM. Ultrasound scans were obtained with the Vevo2100 imaging system (Visual Sonics Inc, Toronto, ON, Canada) with the MS550D probe using a center frequency of 40 MHz. M-mode tracings were measured from the short-axis view. Mitral inflow and tissue velocities were measured using pulsed wave Doppler and tissue Doppler modes from the apical four-chamber view. Mice were carefully monitored following anesthesia to ensure complete recovery. All measurements were obtained from at least three consecutive and consistent cardiac cycles.

**TAIL-CUFF BLOOD PRESSURE MONITORING**

Blood pressure readings were measured using the CODA® tail cuff monitoring system (KentScientific). Mice were first acclimated to the instrument for three consecutive days. Before recording measurements, mice were briefly anesthetized with isoflurane in an induction chamber with 2.5% isoflurane, then restrained, and placed on the warming table. Tail temperatures were monitored and maintained between 32 and 35°C. Data were recorded using the default preferences with five acclimation cycles and ten data collection cycles. Mean arterial pressures were calculated as (SBP+2(DBP))/3.

**INTRAPERITONEAL GLUCOSE TOLERANCE TEST**

Mice were fasted for five hours during the day and then administered a bolus of high glucose solution (125 mg/mL) via intraperitoneal injection (1.25 mg/g body weight). Tail blood was analyzed for glucose levels (mg dL^-1^) and measured using the Contour next EZ (Bayer) blood glucose monitoring system. Blood glucose levels were measured before glucose administration (0 minutes) and then at the following intervals after glucose administration: 15, 30, 45, 60, 90, and 120 minutes.

**SERUM COLLECTION**

Blood Lancets (Medipoint) were used to make a retro-orbital puncture, and blood was collected using capillary serum collection tubes (SAI, MVC-S) in fed mice. Samples were incubated at room temperature for 30 minutes and then centrifuged at 1500 x G for 10 minutes in a refrigerated centrifuge. NEFA was measured using colorimetric enzymatic assays (Wako Diagnostics USA) by the Metabolic Phenotyping Core at UT Southwestern.

**LIPID PROFILING**

Extracted hearts were immediately flash-frozen in liquid nitrogen and then homogenized in ice-cold HPLC-grade isopropanol >10uL/mg tissue. Homogenates were then vortexed for a total of 10 minutes and centrifuged in a 4-degree centrifuge at 14,000 RPM for 10 minutes. The supernatant was then collected and transferred to a clean centrifuge tube and stored in a -80 °C freezer. Positive ion LC-MS/MS was performed by the Proteomics and Metabolomics Core Facility at Weill Cornell Medicine. Intensity values for lipid profiling provided relative quantification comparing the same lipid species across different samples, but not different lipid species compared to one another. Analysis was performed using the MetaboAnalyst 6.0 [1]. Variables with >50% missing values were excluded from analysis and auto-scaled using mean-normalized values. Dot plots and volcano plots were constructed using Scimago Graphic.

**HISTOLOGY**

Hearts or adipose tissue depots were fixed in 10% formalin. Hearts were fixed in formalin overnight, along with interscapular brown tissues, while inguinal and perigonadal depots were fixed for an additional 48 hours. Tissue was then processed using an automated tissue processor for paraffin embedding. Cardiac sections were cut into 7 µm slices, while adipose tissue sections were cut into 5 µm slices. Paraffin sections were incubated at 45°C overnight. Following deparaffinization, slides were incubated in hot citrate buffer (Sigma, C9999) for antigen retrieval. WGA (Invitrogen, W11261) and Isolectin (Invitrogen I21412) were used to determine cardiomyocyte size and vascular density. Picrosirius red stains were prepared by dissolving 0.1 g of Direct Red 80 (Millipore, 365548) into 100 mL of saturated picric acid and then washing with a 0.5% acetic acid solution in DI water. ImageJ ROI tool was used to trace and record cardiomyocyte areas after calibrating the image size to the scale bar. Percentage area measurements for vascular density and fibrosis area were performed by creating RGB stacks for binary images to measure pixels.

For IHC/IF, sections were washed in 3% H_2_O_2_ for 30 minutes after deparaffinization and then blocked in 10% goat serum before overnight incubation with a primary in a 4°C cold room. After washing, sections were incubated in an anti-biotin secondary antibody (Jackson ImmunoResearch, 111-065-144, 1:1000) for two hours, followed by three washes in PBS. Detection using an ABC Peroxidase kit (Thermo, 32020) and Pierce DAB substrate (Thermo, 34002) was carried out according to the manufacturer’s specifications. After development, slides were stained with hematoxylin and mounted with cytosol (Fischer, 8310-16). Images were recorded using Lecia DMi8 with Leica LAS Application Suite X 3.7.4 software.

For Oil Red O stains, fresh hearts are extracted and immediately frozen with O.C.T. compound. Frozen sections were airdried for one hour, briefly rinsed in water, and dipped in 60% isopropyl alcohol before incubating in an Oil Red O staining solution for 10 minutes (Thermo, AAA1298914). Stock Oil Red O (0.3g of Oil Red O in 100mL Isopropanol) was diluted 3:2 in H20 and then filtered to make the staining solution. After incubation in a staining solution, slides were dipped in 60% isopropyl alcohol, rinsed in H20, and then mounted with aqueous mounting media.

**CARDIAC CELLULAR ISOLATION**

Dynabeads were prepared the night prior to EC isolation by mixing (2.5 μg) of ICAM-2 per 2x10^7^ beads on an end-over-end rotator overnight at 4°C. Hearts were then isolated and minced into small pieces and incubated with enzyme solution (Collagenase II; 0.2% in serum-free DMEM) for 50 minutes at 37°C with gentle shaking. The suspension of a 40 μM disposable cell strainer into a 50 mL tube pre-filled with neutralization media (FBS-containing PBS) was centrifuged at 1200 rpm for 8 minutes at 4°C, and then resuspended in the bead washing solution and incubated with ICAM-2 coated Dynabeads for 20 minutes at room temperature with shaking. The bead suspension was then placed on a magnetic rack and washed four times with bead washing solution and once with PBS, and then lysed with RIPA+ protease inhibitor. A Langendorff apparatus was used to isolate cardiomyocytes. Mice were first anesthetized, and then the hearts with intact aorta were harvested. After cannulating the aorta, hearts were perfused with the warmed isolation buffer (130 mM NaCl, 25 mM KCl, 10 mM KH2PO4, 2 mM MgSO4, 10 mM glucose, 20 mM taurine, 5 mM creatine, 100 mM potassium glutamate, 10 mM aspartic acid, 10 mM HEPES, 50 µM CaCl2) and then briefly subjected to further digestion (Type 2 Collagenase). The heart was removed from the Langendorff apparatus and placed in a dissociation buffer (10% FBS and 25 µM Blebbistatin in KB buffer). After removing the atria and right ventricle left ventricular myocytes were dissociated into solution. Calcium was adjusted prior to adding culture media (consisting of MEM supplemented with 25 mM HEPES, 10% FBS, and penicillin/streptomycin). Cardiomyocyte lysates were obtained using RIPA buffer supplemented with protease and phosphatase inhibitors.

**RNA ISOLATION AND QPCR**

Tissues were excised, flash frozen in liquid nitrogen, and then stored in a -80°C freezer. TRIzol was then added to frozen tissues, which were allowed to briefly thaw on ice. Tissues were homogenized using TissueRuptor (Qiagen), centrifuged at 1500xg for two minutes to remove debris, and then isolated using a Direct-zol RNA isolation kit (Zymogen). An additional two rounds of centrifugation for adipose tissues were included to remove lipid contamination, followed by chloroform extraction. RNA concentrations were measured using a BioDrop^TM^. cDNA reactions were prepared using one µg of RNA with MultiScribe™ Reverse Transcriptase, followed by qPCR using SYBR green reagents and a QuantStudio 7 Flex Real-Time PCR system.

**PROTEIN ISOLATION AND WESTERN BLOTTING**

Tissues were excised, flash frozen in liquid nitrogen, and then stored in a -80°C freezer. RIPA buffer with added Halt protease cocktail inhibitor was then added to frozen tissues, which were allowed to thaw on ice briefly. Tissues were homogenized using TissueRuptor (Qiagen) and then centrifuged at 4°C at 1500xg for 15 minutes to remove debris. For adipose tissues, an additional two rounds of centrifugation were performed, and the liquid was then carefully collected to avoid the fat cake, which was separated to prevent lipid contamination. Protein concentrations were then measured using the Pierce Rapid Gold BCA kit. Western blot membranes were blocked in SuperBlock blocking buffer for one hour, incubated in primary antibodies overnight in a 4°C cold room, washed, and incubated with secondary antibodies for two hours, and imaged using an iBright Imaging System (Thermo). When applicable, membranes were stripped with Restore PLUS (Thermo) stripping buffer.

**INDIRECT CALORIMETRY**

Mice were housed individually and acclimatized to the metabolic chambers (Promeethion System, Sable System International) at the UIC Biologic Resources Laboratory for 2 days before data collection was initiated. For the subsequent 3 days, food intake, VO2, VCO2, energy expenditure, and physical activity were monitored over a 12 h light/dark cycle with food provided ad libitum. For energy expenditure analysis, raw data were analyzed using the webserver tool CalR [2]. For body composition analysis, the total fat and lean mass were assessed with Bruker Minispec 10 whole body composition analyzer (Bruker).

**FLOW CYTOMETRY**

Sample collection was performed at consistent times for each group. Peripheral blood was obtained via lateral facial vein puncture. Mice were euthanized and perfused via the left ventricle with ice-cold phosphate-buffered saline (PBS) to remove circulating blood. Adipose tissues were collected and briefly minced with scissors and then placed into a conical vial on ice with digest buffer (1xHBSS, 3%BSA, Collagenase II [288U/mL], CaCl_2_[1M], MgCl_2_[1M], ZnCl_2_[0.1M], ddH_2_0). Samples were briefly vortexed to promote dissociation, and then incubated at 37°C for 15 minutes on a shaker, and then briefly vortexed once again and incubated for another 15 minutes. Digested cells were then centrifuged at 200xg for 5 minutes at 4 °C, resuspended in RPMI+10% FBS, and centrifuged at 200xg. Pellets were then resuspended in ACK lysis buffer and incubated for 2 minutes for RBC lysis, then quenched with RMPI+10% FBS. The suspension was then filtered through a 40 µM filter placed in a clean conical vial and then centrifuged at 200xg for 5 minutes at 4 °C. Pellets were then resuspended in FACS buffers (EDTA [10mM], 0.5% BSA, PBS) and labeled with the indicated fluorophore-conjugated antibodies and analyzed on a BD FACSCelesta flow cytometer (BD Bioscience) with FACSDIVA software (BD Biosciences). Data were analyzed using FlowJo software (FlowJo, LLC).

**TRANSPLANTATION STUDIES**

All surgical procedures were performed in compliance with our ACC-approved protocol. Mice were administered meloxicam (5mg/kg daily I.P. injections for 2 days) as an analgesic. Anesthesia was induced as described above. A warming platform was used to avoid heat loss during surgery. Adipose tissue depots from donor mice were excised and briefly washed in a warm sterile saline solution before being transferred to recipient mice. Prior to incision, surgical sites were cleaned in Betadine, and then abdominal cavities were opened with sterilized surgical tools. Recipient mice were given approximately 100 mg of iBAT or iWAT from CL-treated donors and carefully placed within the fold of the gWAT. For sham-treated mice, gWAT tissue was exposed and returned to its original position. The body cavities were then sealed with surgical sutures. Mice were then carefully monitored following the procedure for any signs of infection, distress, or discomfort.

**STATISTICAL ANALYSES**

Statistical significance was determined using Prism software or Excel. The values are presented as the mean ± standard deviation (SD) or standard error of the mean (SEM) as indicated by the figure legends. Statistical differences were determined by a 2-tailed, unpaired Student’s t test or one-way ANOVA with Tukey’s multiple comparison test as appropriate. Values of p < 0.05 were considered statistically significant. Statistical analysis of lipidomic data were performed using <https://www.metaboanalyst.ca/> and graphed using scimago graphica.

**Antibodies & Reagents**

| Wheat Germ Agglutinin, Alexa Fluor 488 Conjugate | Invitrogen | W11261 |
| --- | --- | --- |
| 4/80 Monoclonal Antibody (BM8), APC | Invitrogen | 17-4801-80 |
| Acetyl Lysine Monoclonal Antibody (1C6), Unconjugated | Invitrogen | MA1-2021 |
| Acetyl-CoA Carboxylase Polyclonal Antibody | Invitrogen | PA5-17564 |
| Alexa Fluor 488 donkey anti-rabbit IgG | Invitrogen | A21206 |
| Alexa Fluor Plus 555 donkey anti-rat IgG | Invitrogen | A48270 |
| ATP Citrate Lyase Antibody | Invitrogen | MA5-24861 |
| Beta-1 Adrenergic Receptor | Invitrogen | PA1-049 |
| Beta-2 Adrenergic Receptor | Invitrogen | PA5-14117 |
| Beta-3 Adrenergic Receptor | Invitrogen | PA5-50914 |
| beta-3 Tubulin Monoclonal Antibody (2G10) | Invitrogen | MA1-118X |
| Cardiac Troponin T | Abcam | ab45932 |
| Cd102 (ICAM-2) Monoclonal Antibody (3C4 (mIC2/4)) | Invitrogen | 16-1021-82 |
| CD11b Monoclonal Antibody (M1/70), APC-Cyanine7 | Invitrogen | A15390 |
| Cd11c-PE; Clone: N418 | Invitrogen | 12-0114-81 |
| Cd206 (MMR) Monoclonal Antibody (MR6F3), PE-Cyanine7 | Invitrogen | 25-2061-80 |
| CD31 Polyclonal Antibody | Invitrogen | PA5-16301 |
| CD3e Monoclonal Antibody (145-2C11), PerCP-Cyanine5.5 | Invitrogen | 45-0031-80 |
| Cd4 Monoclonal Antibody (GK1.5), PE, eBioscience™ | Invitrogen | 12-0041-81 |
| Cd45-FITC, eBioscience™ | Invitrogen | 11-0451-81 |
| CD45R (B220) Monoclonal Antibody (RA3-6B2) | Invitrogen | 17-0452-82 |
| Cd8a Monoclonal Antibody (53-6.7), PE-Cyanine7, | Invitrogen | 25-0081-81 |
| Collagenase type II | MP Bio | 100502 |
| Dynabeads sheep anti-Rat IgG, cat. No. 11035 | Invitrogen | 11035 |
| eNOS | Invitrogen | PA1-037 |
| FASN | Invitrogen | PA522061 |
| GAPDH | Abcam | ab8245 |
| IB4 From Griffonia simplicifolia, Alexa Fluor 568 Conjugate | Invitrogen | I21412 |
| iNOS | Invitrogen | PA1-036 |
| nNOS | Invitrogen | 61-7000 |
| Perilipin 1 Monoclonal Antibody | Invitrogen | PA5-72921 |
| phospho-eNOS (Ser1177) | Invitrogen | MA5-14957 |
| Phospho-eNOS (Thr495) | Invitrogen | PA5-17706 |
| Phospho-PKA substrate | Cell signaling | 9624S |
| UCP1 Polyclonal | Invitrogen | PA1-24894 |
| Vinculin Recombinant Rabbit Monoclonal Antibody | Invitrogen | 700062 |

**PCR Primers**

| **Gene** | **Forward** | **Reverse** |
| --- | --- | --- |
| Col1a1 | CCTCAGGGTATTGCTGGACAAC | CAGAAGGACCTTGTTTGCCAGG |
| Col1a2 | TTCTGTGGGTCCTGCTGGGAAA | TTGTCACCTCGGATGCCTTGAG |
| Ctgf | TGCGAAGCTGACCTGGAGGAAA | CCGCAGAACTTAGCCCTGTATG |
| Adrb1 | AGTGCTGCGATTTCGTCACCAACA | GCTCGCAGCTGTCGATCTTCTTTA |
| Adrb2 | TGCTATCACATCGCCCTTC | ACCACTCGGGCCTTATTCTT |
| Adrb3 | CAGCCAGCCCTGTTGAAG | CCTTCATAGCCATCAAACCTG |
| Prdm16 | ATCCACAGCACGGTGAAGCCAT | ACATCTGCCCACAGTCCTTGCA |
| Ucp1 | GCTTTGCCTCACTCAGGATTGG | CCAATGAACACTGCCACACCTC |
| Fasn | CACAGTGCTCAAAGGACATGCC | CACCAGGTGTAGTGCCTTCCTC |
| Acc2 | AGAAGCGAGCACTGCAAGGTTG | GGAAGATGGACTCCACCTGGTT |
| Cidea | GGTGGACACAGAGGAGTTCTTTC | CGAAGGTGACTCTGGCTATTCC |
| Dio2 | GGTGGTCAACTTTGGTTCAGCC | AAGTCAGCCACCGAGGAGAACT |
| Pgc1α | GAATCAAGCCACTACAGACACCG | CATCCCTCTTGAGCCTTTCGTG |
| Chrebp | GAGTGCTTGAGCCTGGCTTACA | GCTCTCCAGATGGCGTTGTTCA |
| Acly | AGGAAGTGCCACCTCCAACAGT | CGCTCATCACAGATGCTGGTCA |
| Scd1 | GCAAGCTCTACACCTGCCTCTT | CGTGCCTTGTAAGTTCTGTGGC |
| Pnpla2 | GGAACCAAAGGACCTGATGACC | ACATCAGGCAGCCACTCCAACA |

**Supplemental Table 1. Echocardiographic parameters were measured in different groups of experimental mice.** Results from echocardiographic parameters measured from m-mode traces and apical four-chamber views obtained to evaluate systolic and diastolic function in unconscious mice from the indicated groups following HFpEF treatment; *n* = 10 mice per group; Data are presented as the mean ± SEM; Significance values were determined by one-way analysis of variance (ANOVA) followed by Tukey’s multiple comparisons; *P ≤ 0.05, **P ≤ 0.01, ***P ≤ 0.001.

**REFERENCES**

1. Pang, Z., et al., *MetaboAnalyst 5.0: narrowing the gap between raw spectra and functional insights.* Nucleic Acids Research, 2021. **49**(W1): p. W388-W396.

2. Mina, A.I., et al., *CalR: A Web-Based Analysis Tool for Indirect Calorimetry Experiments.* Cell Metabolism, 2018. **28**(4): p. 656-666.e1.

**
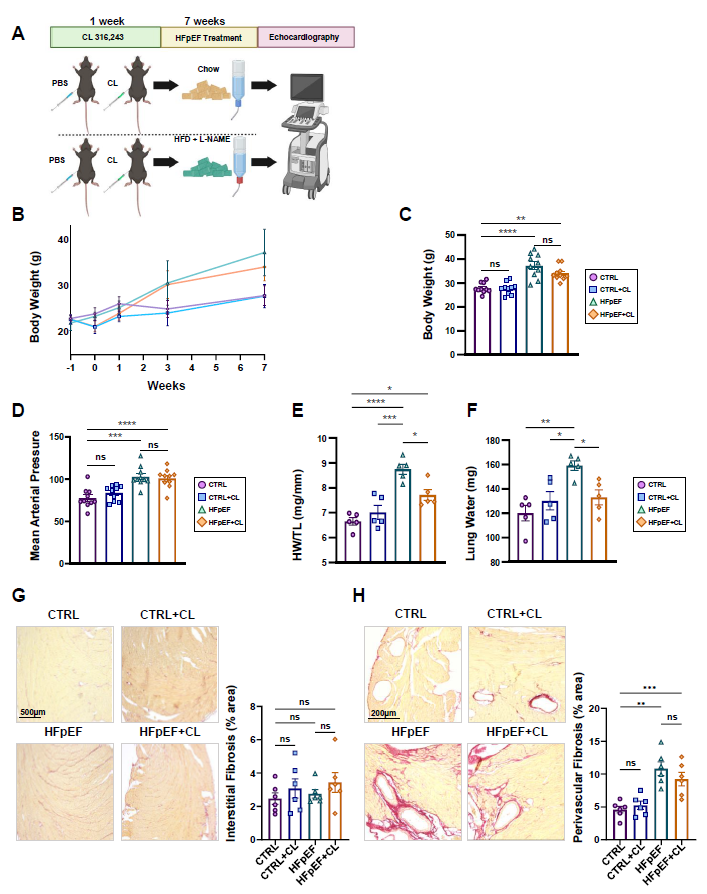
**

**Supplemental Figure 1. Characterization of cardiac pathological manifestations developed during HFpEF treatment.** The experimental workflow shows vehicle or CL treatment followed by chow diet or HFpEF treatment. At the experimental endpoint, echocardiography is used to evaluate cardiac function (A). Body weights before and after CL treatment and after the seven-week treatment period (B, C). Mean arterial pressures recorded using the CODA tail-cuff monitoring system in acclimatized mice (D); n = 10 mice per group. Heart weight to tibia length measurements were obtained following HFpEF treatment (E); n = 5 per group. Total lung water was calculated as the difference between wet lung and dry lung weights recorded after HFpEF treatment (F); n = 5 per group. Picrosirius red-stained hearts with quantification of the percentage of positively stained area per field of view as measured across interstitial tissue (G), and in areas defined as perivascular regions (H); Scale bars are equal to 500 µm (G) or 200 µm (H); n = 6 mice per group. Significance values are determined by a one-way analysis of variance (ANOVA) followed by Tukey’s multiple comparisons: *P ≤ 0.05, **P ≤ 0.01, ***P ≤ 0.001, ****P ≤ 0.0001.

**
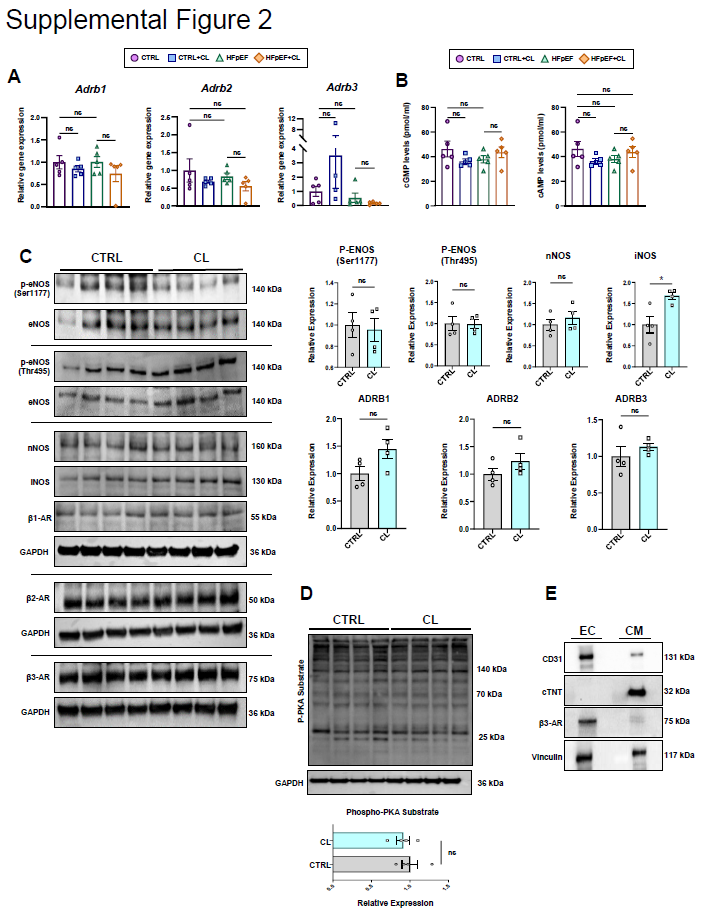
**

**Supplemental Figure 2.** **Molecular characterization of the β3-adrenergic pathway in cardiac tissues**. Gene expression analysis of beta-adrenergic receptors (β1-β3), in cardiac extracts, determined by RT-qPCR (**A**); *n* = 5 per group. Competitive ELISA determined cGMP or cAMP concentration from cardiac tissue extracts (**B**); *n* = 5 per group. Western blotting results obtained from cardiac extracts collected immediately following CL treatment evaluate the expression of nitric oxide synthases and phosphorylation status at phospho-active/ inhibitory residues, as well as β-adrenergic receptors (β1-β3), with black lines indicating separate gels (**C**). PKA activity as measured by the total amount of phosphorylated PKA substrates in cardiac extracts by western blotting (**D**); *n* = 4 per group. Cellular isolation obtained from hearts showing endothelial or cardiomyocytes expression of β3-AR, along with cellular-specific markers to validate isolation purity (**E**). Data are presented as the mean ± SEM. Significance values were determined by one-way analysis of variance (ANOVA) followed by Tukey’s multiple comparisons (**A, B**) or by Two-tailed unpaired Student’s *t*-test (**C, D**); *P ≤ 0.05.


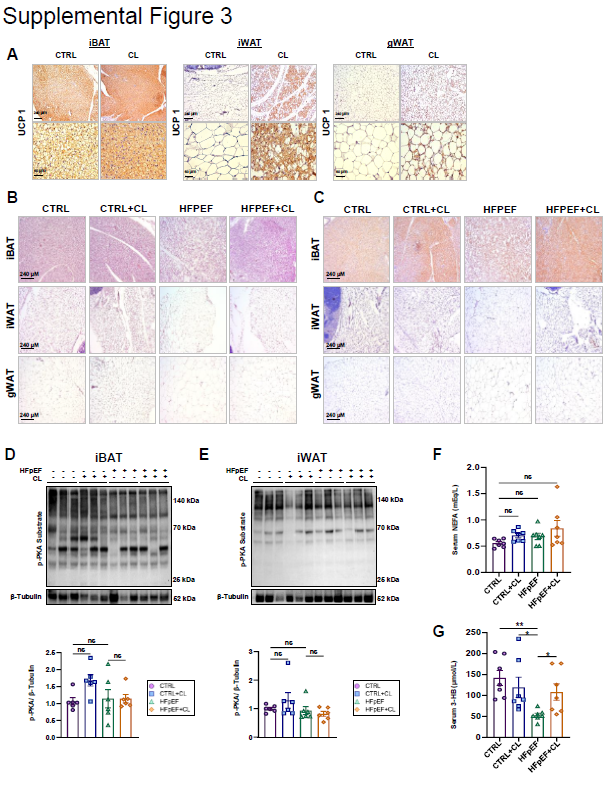


**Supplemental Figure 3. Histological and molecular characterization of CL-treated adipose tissues after HFpEF treatment.** Representative images of adipose tissue remodeling occurring in the acute period, immediately after treatment with CL, showing the increased presence of multilocular adipocytes and enhanced expression of UCP1 shown by Immunohistochemical (IHC) in the interscapular brown adipose tissue (iBAT), inguinal white adipose tissue (iWAT), and gonadal white adipose tissue (gWAT) (**A**). Representative images of adipose tissues sectioned from iBAT, iWAT, gWAT of either CTRL, CTRL+CL, HFpEF or HFpEF+CL mice assessed by Hematoxylin and Eosin (H&E) staining and IHC staining for UCP1 (**B**, **C**); Image magnifications were recorded using a 5X objective; Scale bars equal 240 µm. PKA activity measured as the total amount of phosphorylated PKA substrates measured in proteins isolated from iBAT (**D**) or iWAT (**E**) determined by western blotting; *n* = 6 per group. Serum concentrations of non-esterified fatty acids (NEFA) (**F**) or beta-hydroxybutyrate (BHB) (**G**) measured by colorimetric assay following HFpEF treatment; *n* = 7 mice per group. Data are presented as the mean ± SEM; Significance values were determined by one-way analysis of variance (ANOVA) followed by Tukey’s multiple comparisons. *P ≤ 0.05, **P ≤ 0.01, ***P ≤ 0.001, ****P ≤ 0.0001.

**
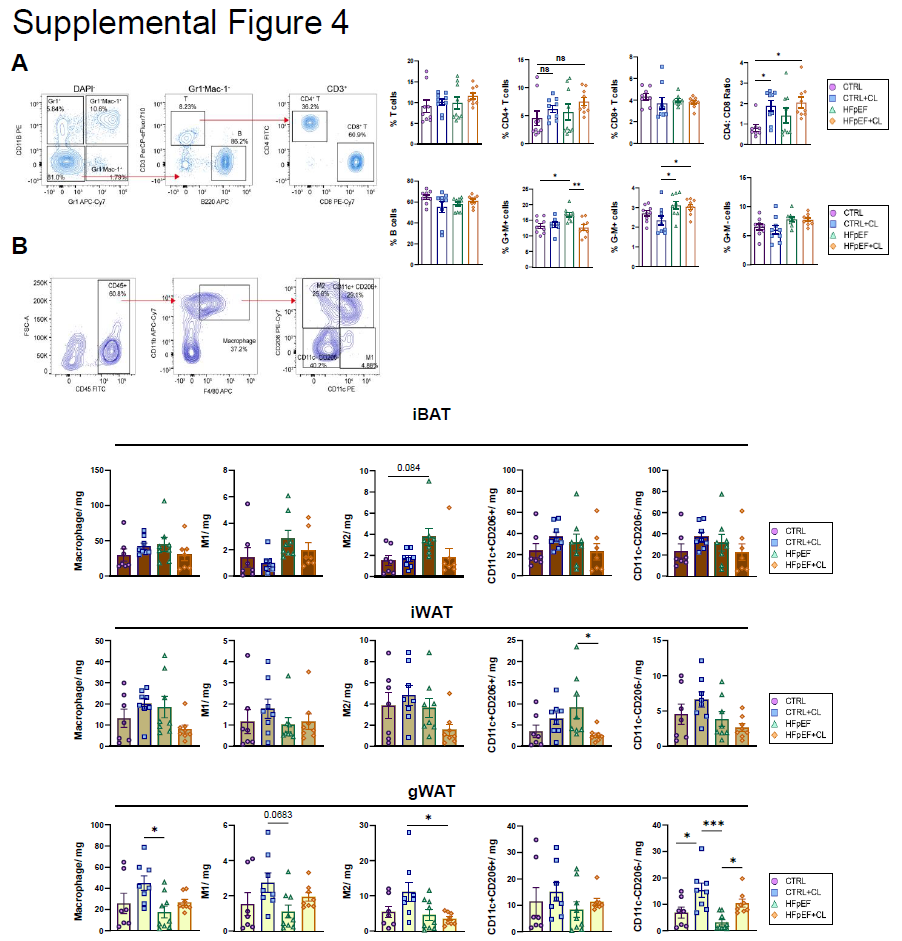
**

**Supplemental Figure** **4. Profiling of changes in circulating and adipose tissue immune cells.** Analysis of peripheral blood (PB) by Fluorescence-activated cell sorting (FACS) using the indicated gating strategy shown in the diagram to measure for changes in T-cell subsets CD4+ and CD8+ T-cells, B-cells, and populations of G+M+ cells (**A**); n=9 per group. Analysis of iBAT, iWAT, and gWAT using FACS identified changes in M1 and M2 macrophage populations using CD11c and CD206, as shown in the gating strategy, with data points representing the cell count normalized to tissue weights (**B**); n=8 per group. Significance values are determined by a one-way analysis of variance (ANOVA) followed by Tukey’s multiple comparisons: *P ≤ 0.05, **P ≤ 0.01, ***P ≤ 0.001.


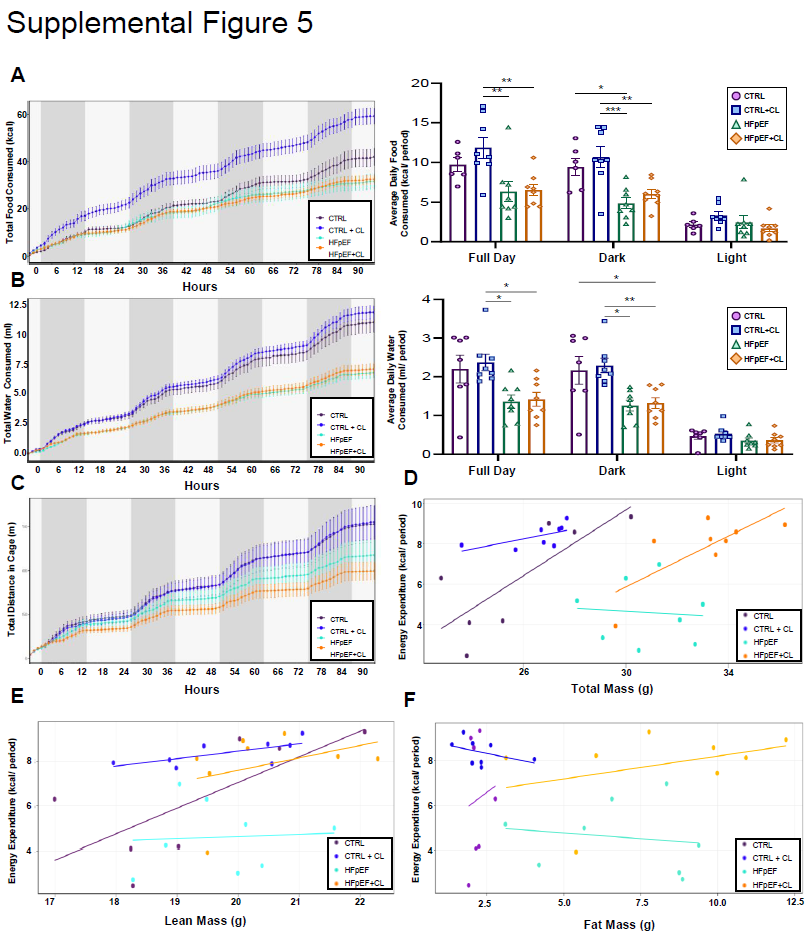


**Supplemental Figure 5. Metabolic parameters recorded during metabolic cage experiments.** Total food consumed along with average daily consumption values (**A**), total water consumed along with average daily consumption values over a given activity period (**B**), and total distance traveled (**C**) for each experimental group are displayed as line charts over the indicated periods recorded in hours or in box plots where aggregated values are reported as the mean value for a defined time frame (Full day, Dark, Light). Regression analysis shows the correlation between energy expenditure values and total mass (**D**), lean mass (**E**), and fat mass (**F**). Data are presented as the mean ± SEM; *n* = 8 mice per group; Significance values are determined by a one-way analysis of variance (ANOVA) followed by Tukey’s multiple comparisons: *P ≤ 0.05, **P ≤ 0.01, ***P ≤ 0.001.


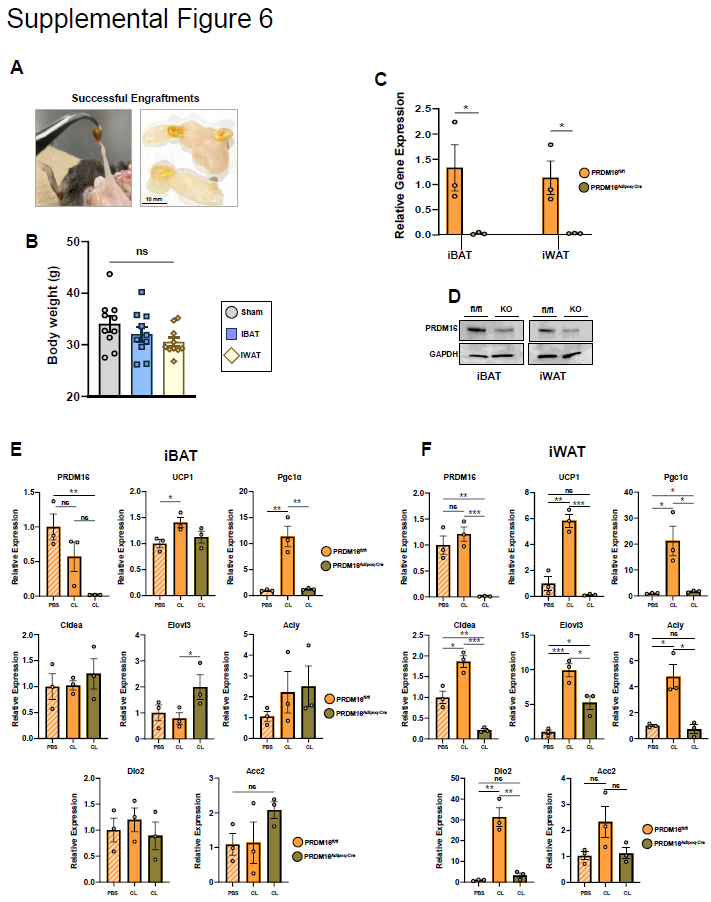


**Supplemental Figure 6. Direct transplantation or disruption of thermogenic adipose tissue function determines the cardioprotective effects of CL treatment.** Representative images of successful adipose tissue engraftments following transplantation (**A**). Body weights were measured in sham-operated, iBAT, and iWAT transplant recipient mice after HFpEF treatment (**B**); *n* = 10 mice per group. Gene expression analysis of *Prdm16* levels in the iBAT and iWAT of PRDM16-KO or floxed controls as determined by RT-qPCR, measuring mice. *n* = 3 mice per group (**C**). Protein expression of PRDM16 in the iBAT and iWAT from PRDM16-KO mice or floxed controls (**D**). Gene expression changes determined by RT-qPCR surveying a panel of thermogenic markers in PRDM16-KO mice or floxed controls after receiving treatment with either PBS or CL show the severely blunted response PRDM16-KO mice have to CL treatment; *n* = 3 mice per group. Data are presented as the mean ± SEM; Significance values were determined by Two-tailed unpaired Student’s *t*-test: *P ≤ 0.05, **P ≤ 0.01, ***P ≤ 0.001.


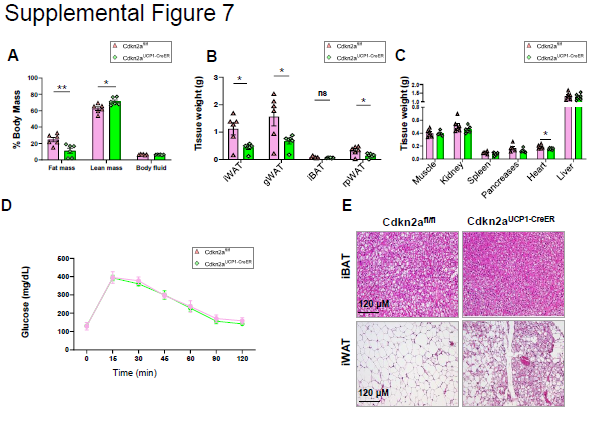


**Supplemental Figure 7. Characterization of Cdkn2a^UCP1-CreER^ KO** **mice exposed to HFpEF conditions.** Body mass compositions as determined by nuclear magnetic resonance (**A**); *n* = 6 per group. Individual fat pad weights for iWAT, gWAT, iBAT and retroperitoneal adipose tissue (rpWAT) (**B**); *n* = 6 per group. Tissue weights obtained from the indicated organs (**C**); *n* = 6 per group. Results from a fasting glucose tolerance test were recorded at the end of the HFpEF treatment (**D**); *n* = 6 per group. Representative H&E staining results of iBAT and iWAT from Cdkn2a^UCP1-CreER^ KO mice or floxed controls (HFpEF) (**E**); Image magnifications are recorded using 10X objective; Scale bars equal 120 µm; Data are presented as the mean ± SEM; A Two-tailed unpaired Student’s t-test determines significance values; *P ≤ 0.05, **P ≤ 0.01.


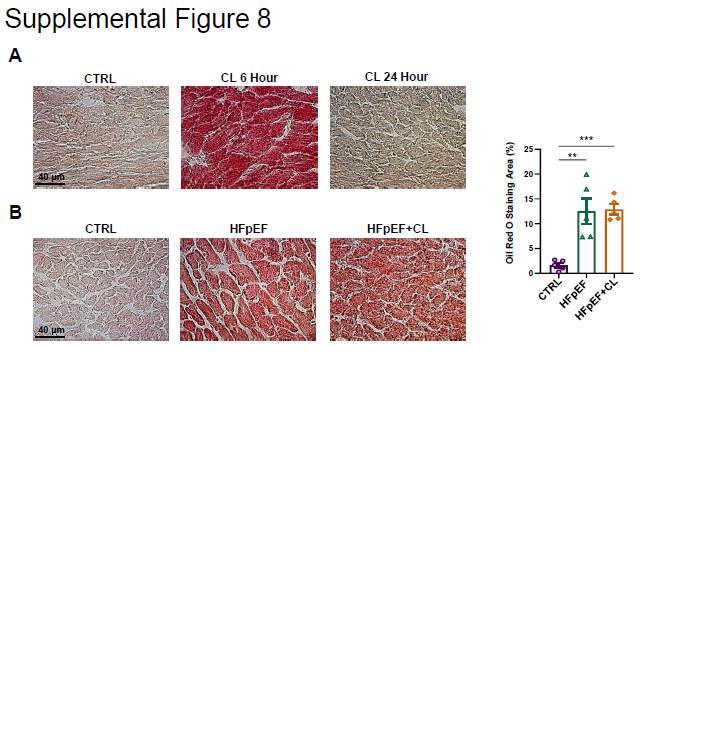


**Supplemental Figure 8. Cardiac lipid uptake is increased following the administration of CL.** Oil Red O staining (ORO) staining of cardiac extracts collected from wild-type mice under basal conditions and then at six and twenty-four hours after administration of CL (**A**). ORO staining in cardiac extracts of CTRL, HFpEF, and HFpEF+CL mice collected after HFpEF treatment (**B**). Image magnifications are recorded using 63X objective; Scale bars equal 40 µm; Significance values are determined by one-way analysis of variance (ANOVA) followed by Tukey’s multiple comparisons; *P ≤ 0.05, **P ≤ 0.01, ***P ≤ 0.001.


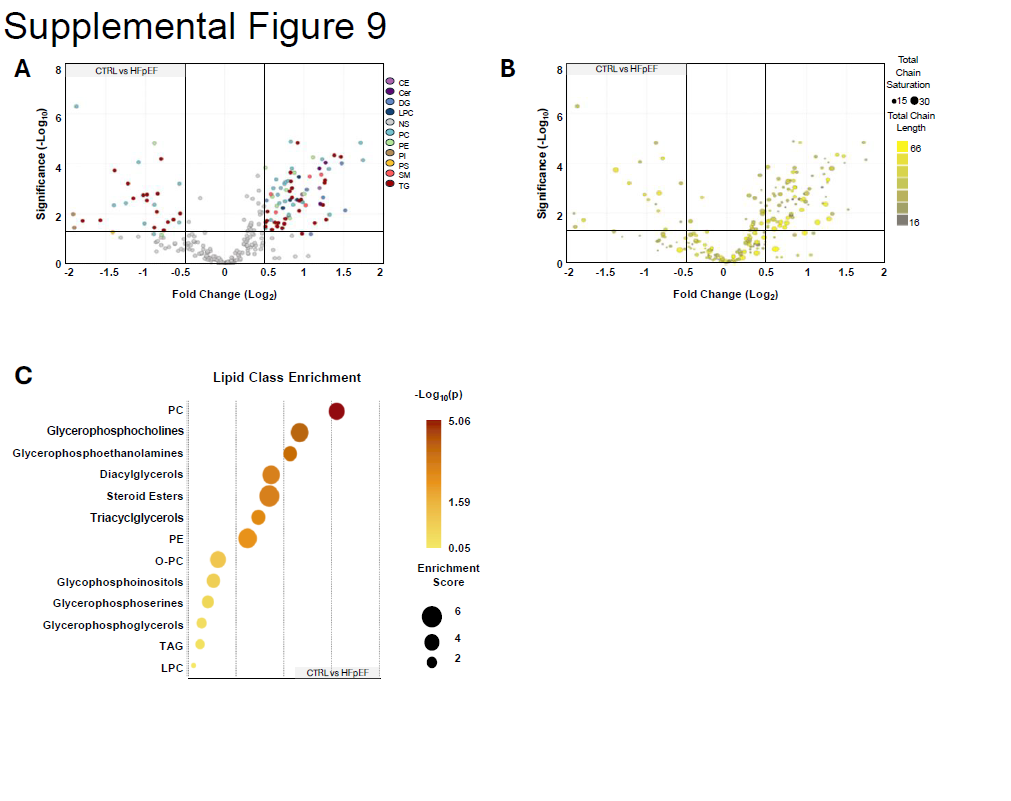


**Supplemental Figure 9. Lipidomic profiling reveals differences in cardiac lipid composition among different treatments.** Volcano plots display significance and log_2_fold change (Log_2_FC > ± 0.5, p< 0.05) in the relative differences in lipid quantities. Data points are colored according to lipid classes or total chain saturation level, where total saturation levels are weighted by the size of the data point as indicated by the legend. Volcano plots display alterations in lipid composition which occur as a result of HFpEF treatment by comparing the changes that occur between HFpEF and CTRL (**A**, **B**). Quantitative enrichment analysis results show the lipid classes that are most significantly affected by HFpEF treatment and are colored according to signfance values and weighed according to enrichment scores as shown in the diagram (**C**).


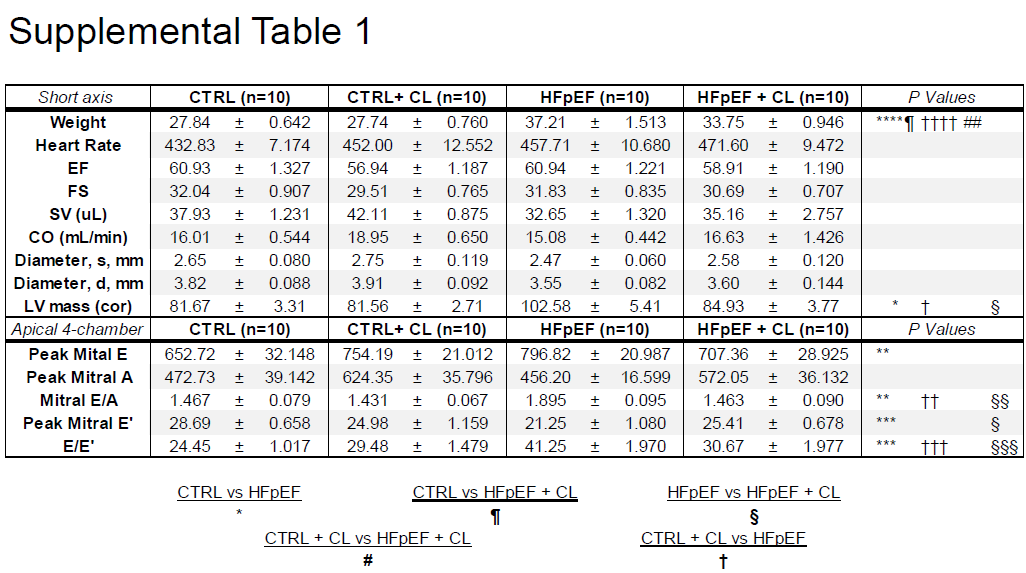


**Supplemental Table 1. Echocardiographic parameters were measured in different groups of experimental mice.** Results from echocardiographic parameters measured to evaluate systolic and diastolic function in unconscious mice from the indicated groups following HFpEF treatment; *n* = 10 mice per group; Data are presented as the mean ± SEM; Significance values were determined by one-way analysis of variance (ANOVA) followed by Tukey’s multiple comparisons; *P ≤ 0.05, **P ≤ 0.01, ***P ≤ 0.001.
